# Supplementary material for: ERK Signaling Pathway Is Constitutively Active in NT2D1 Non-Seminoma Cells and Its Inhibition Impairs Basal and HGF-Activated Cell Proliferation
Source: Biomedicines. 2023 Jul 4;11(7):1894. doi: 10.3390/biomedicines11071894 (PMC10377482; doi:10.3390/biomedicines11071894)
Supplement: Supplementary file 1 [file biomedicines-11-01894-s001.zip › Figure S1 Gesualdi et al., biomedicines.pdf]

Figure S.1. UO126 dose response experiment.

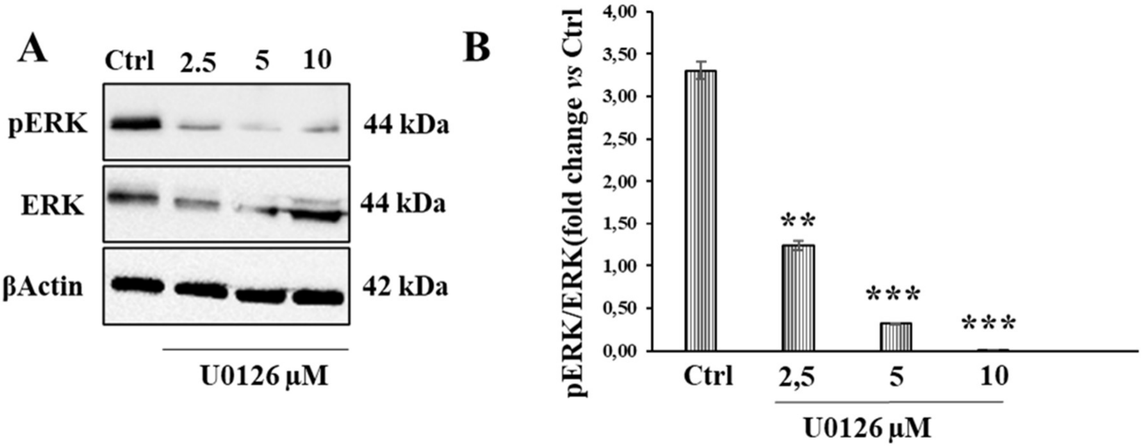

Figure S.1. (**A**) Western Blot analyses of phospho- and total ERK1/2 performed on cells cultured in basal condition and 30 minutes after U0126 administration U0126 (2.5, 5, 10 μM). (**B**) Graphical representation of densitometric analyses of the bands (2.5 μM) ** *vs* CTRL p≤0.01. (5,10 μM) *** *vs* CTRL p≤0.001. Results were expressed in fold change, with the control considered as 1 (±standard error of the mean (S.E.M)).
