# Supplementary material for: ERK Signaling Pathway Is Constitutively Active in NT2D1 Non-Seminoma Cells and Its Inhibition Impairs Basal and HGF-Activated Cell Proliferation
Source: Biomedicines. 2023 Jul 4;11(7):1894. doi: 10.3390/biomedicines11071894 (PMC10377482; doi:10.3390/biomedicines11071894)
Supplement: Supplementary file 1 [file biomedicines-11-01894-s001.zip › Figure S2 Gesualdi et al., biomedicines.pdf]

Figure S.2. Cell cycle analysis of T0 and 48 h cultured cells with or without UO126.

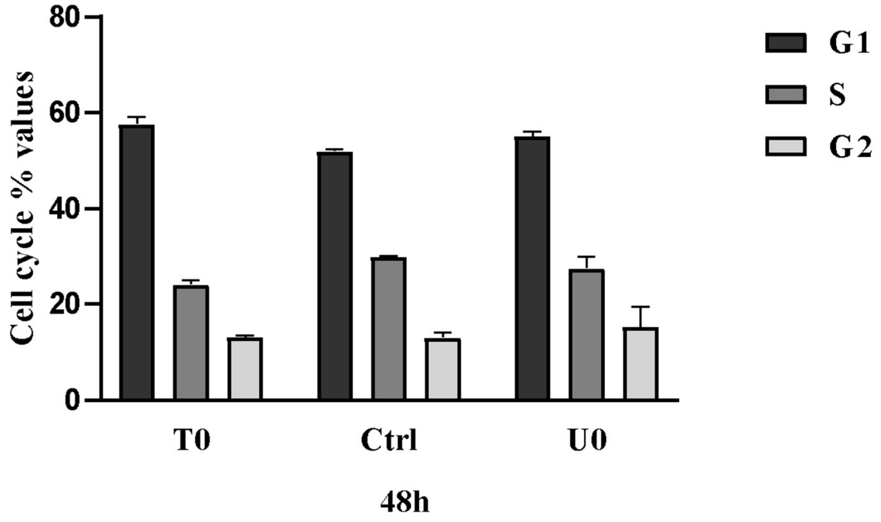

Figure S.2. Graphical representation of cell cycle analysis on NT2D1 cell cultured for 48 h with or without U0126 inhibitor, illustrating cell % for each cell cycle phase in all the experimental conditions reported in the respective graph ± S.E.
