# Supplementary material for: ERK Signaling Pathway Is Constitutively Active in NT2D1 Non-Seminoma Cells and Its Inhibition Impairs Basal and HGF-Activated Cell Proliferation
Source: Biomedicines. 2023 Jul 4;11(7):1894. doi: 10.3390/biomedicines11071894 (PMC10377482; doi:10.3390/biomedicines11071894)
Supplement: Supplementary file 1 [file biomedicines-11-01894-s001.zip › Figure S3 Gesualdi et al., biomedicines V2.pdf]

Figure S.3.A. Whole western blots.

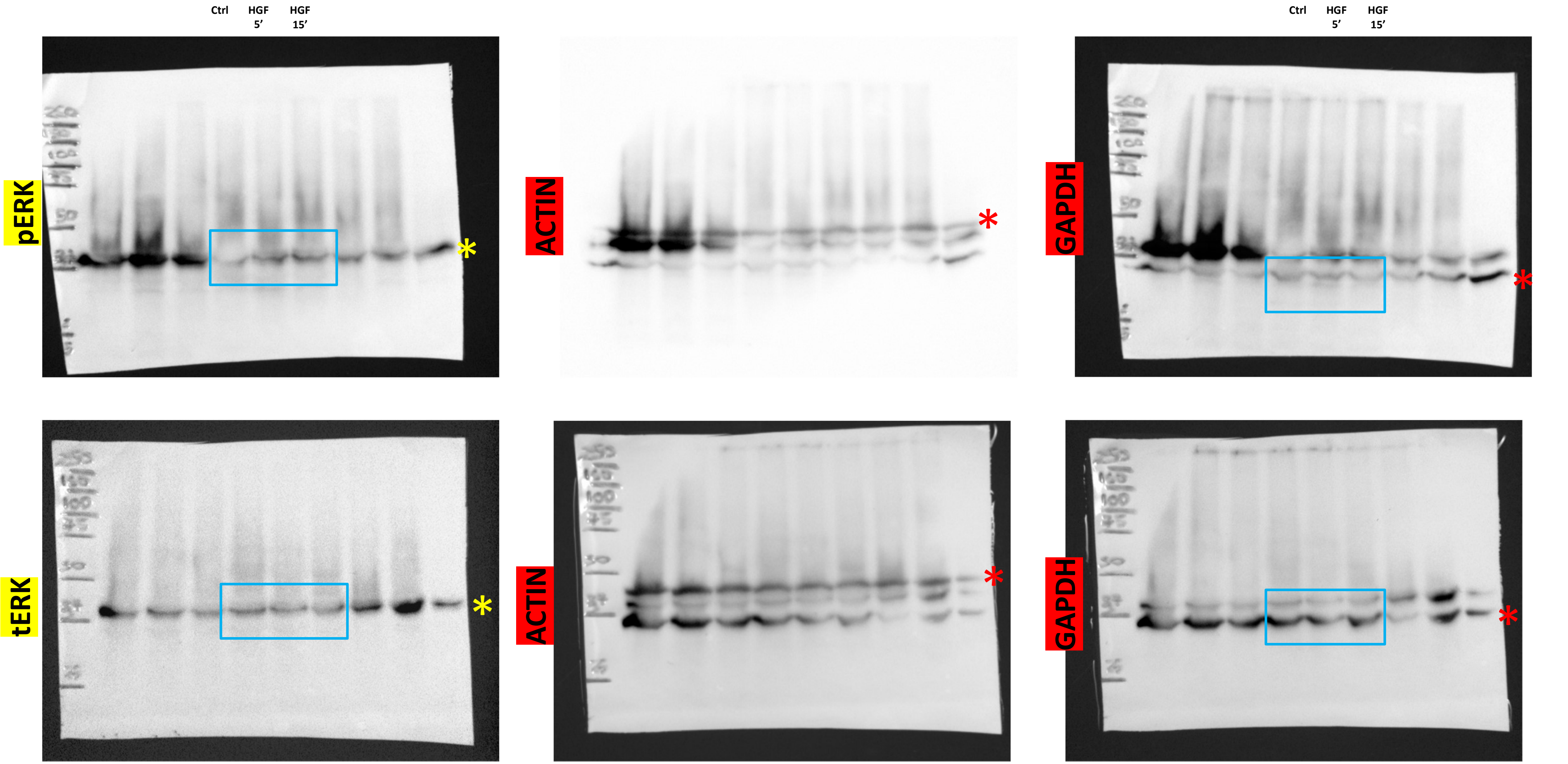

Blue boxes indicate WB lines selected for figures included in the paper.
FIGURE 1A

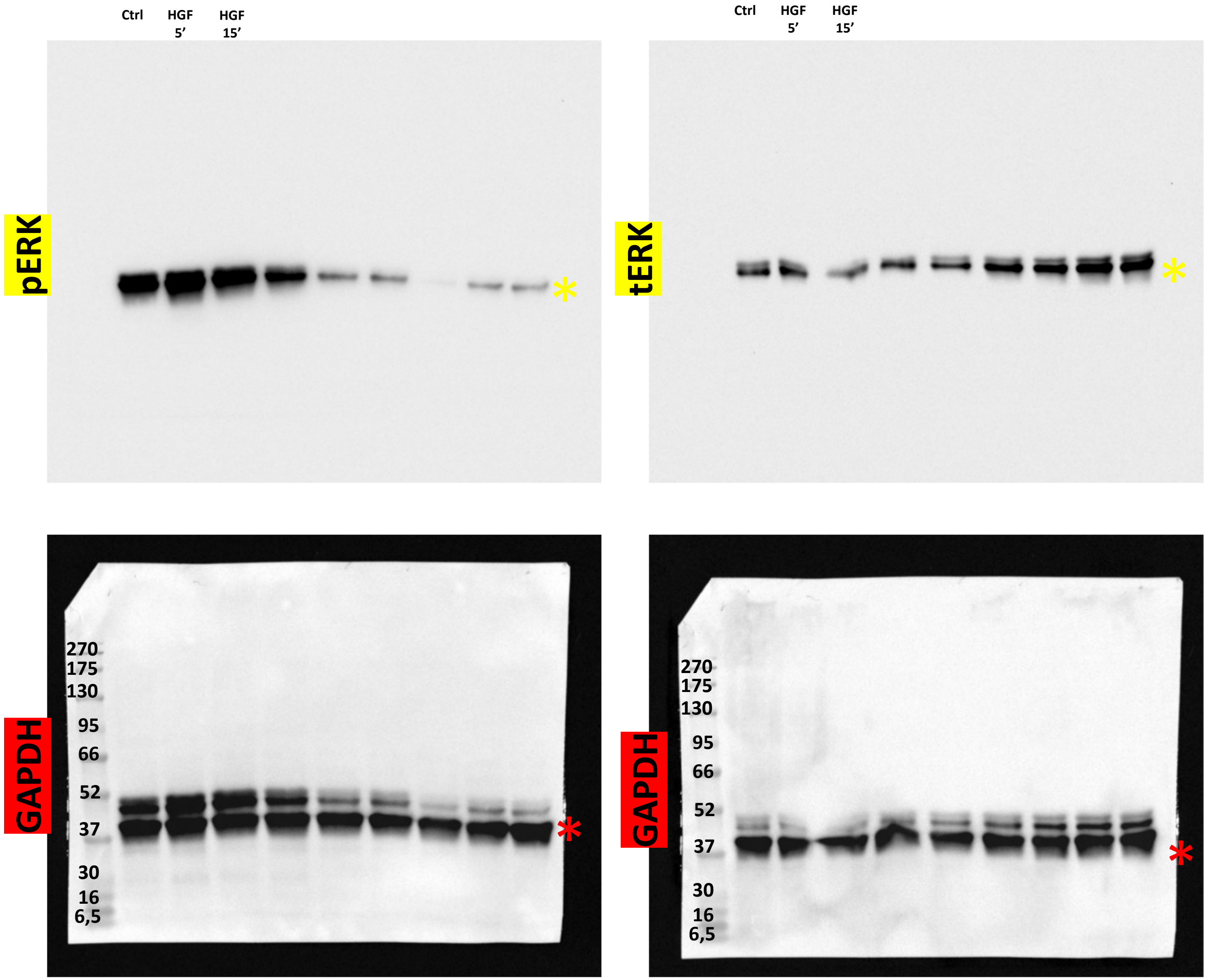

These blots are relative to results presented in FIGURE 1

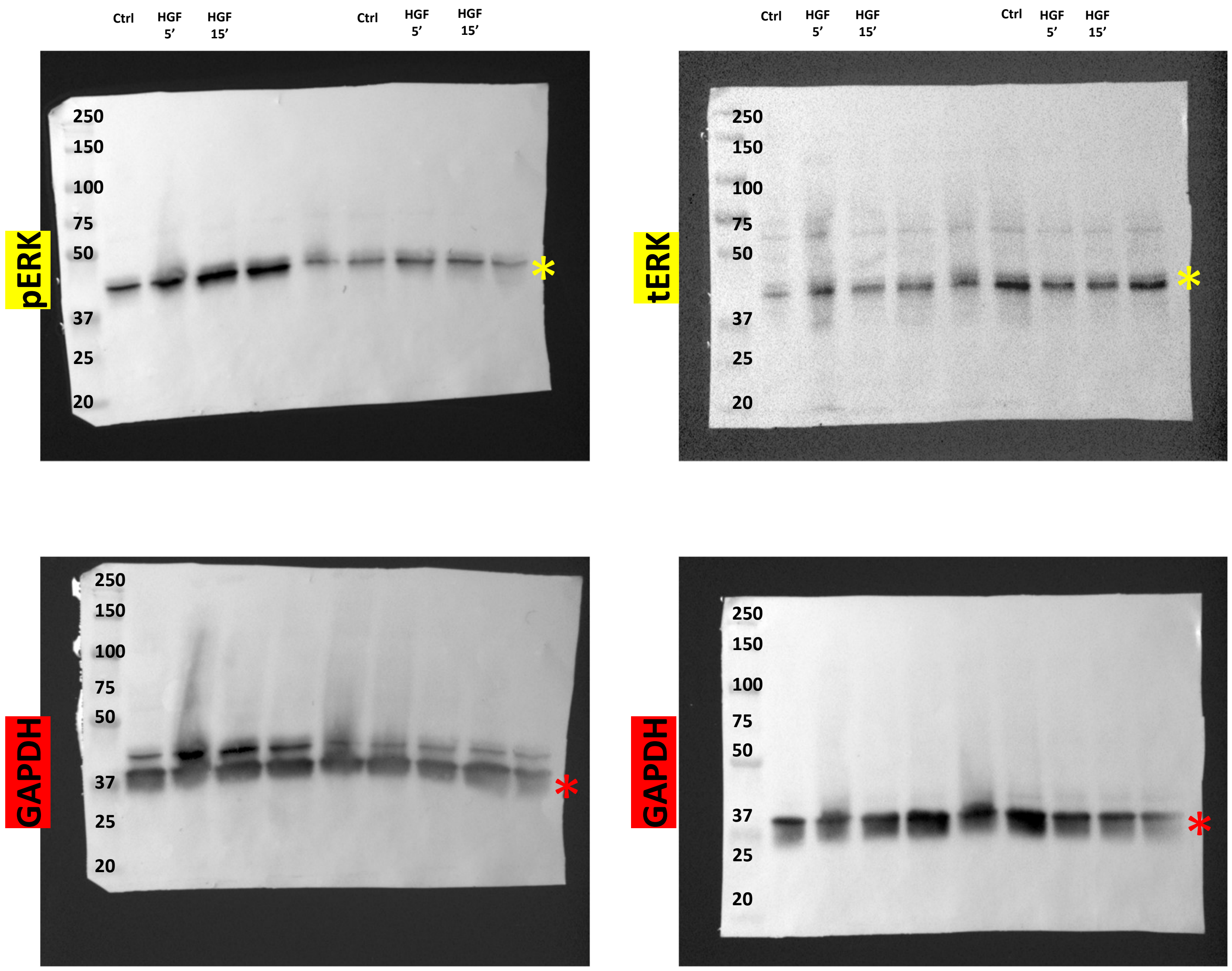

These blots are relative to results presented in FIGURE 1

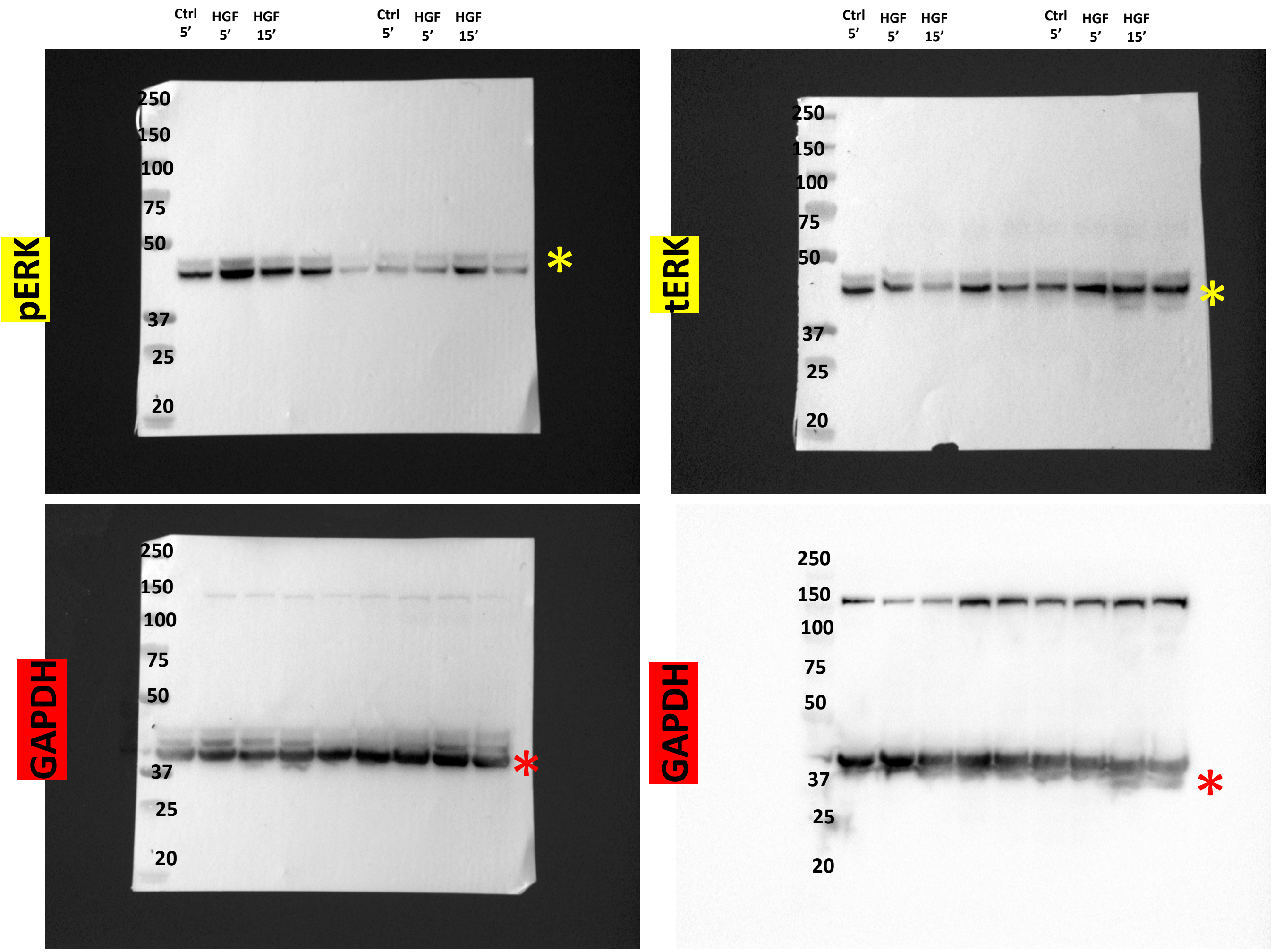

These blots are relative to results presented in FIGURE 1

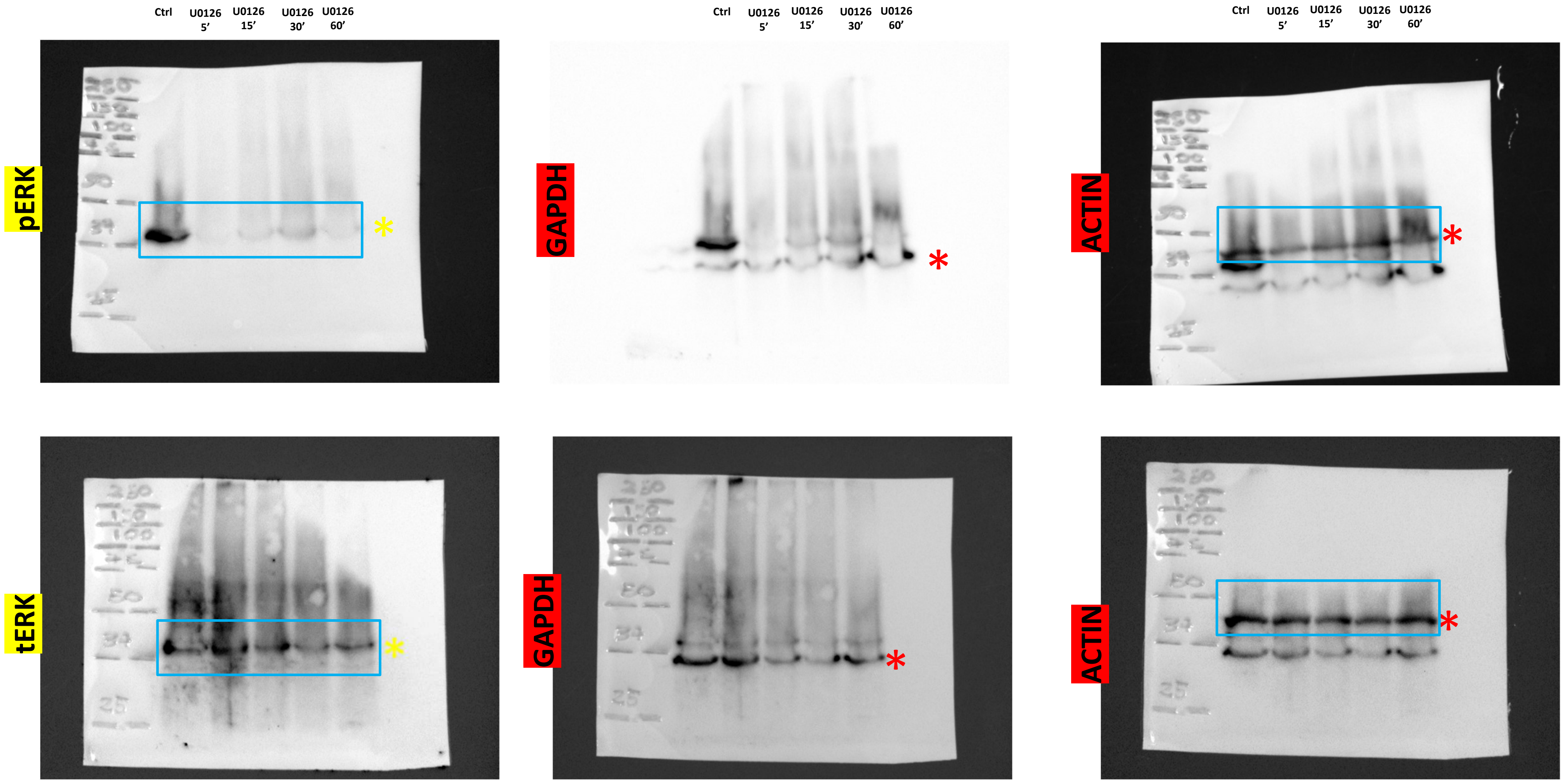

Blue boxes indicate WB lines selected for figures included in the paper.
FIGURE 2B

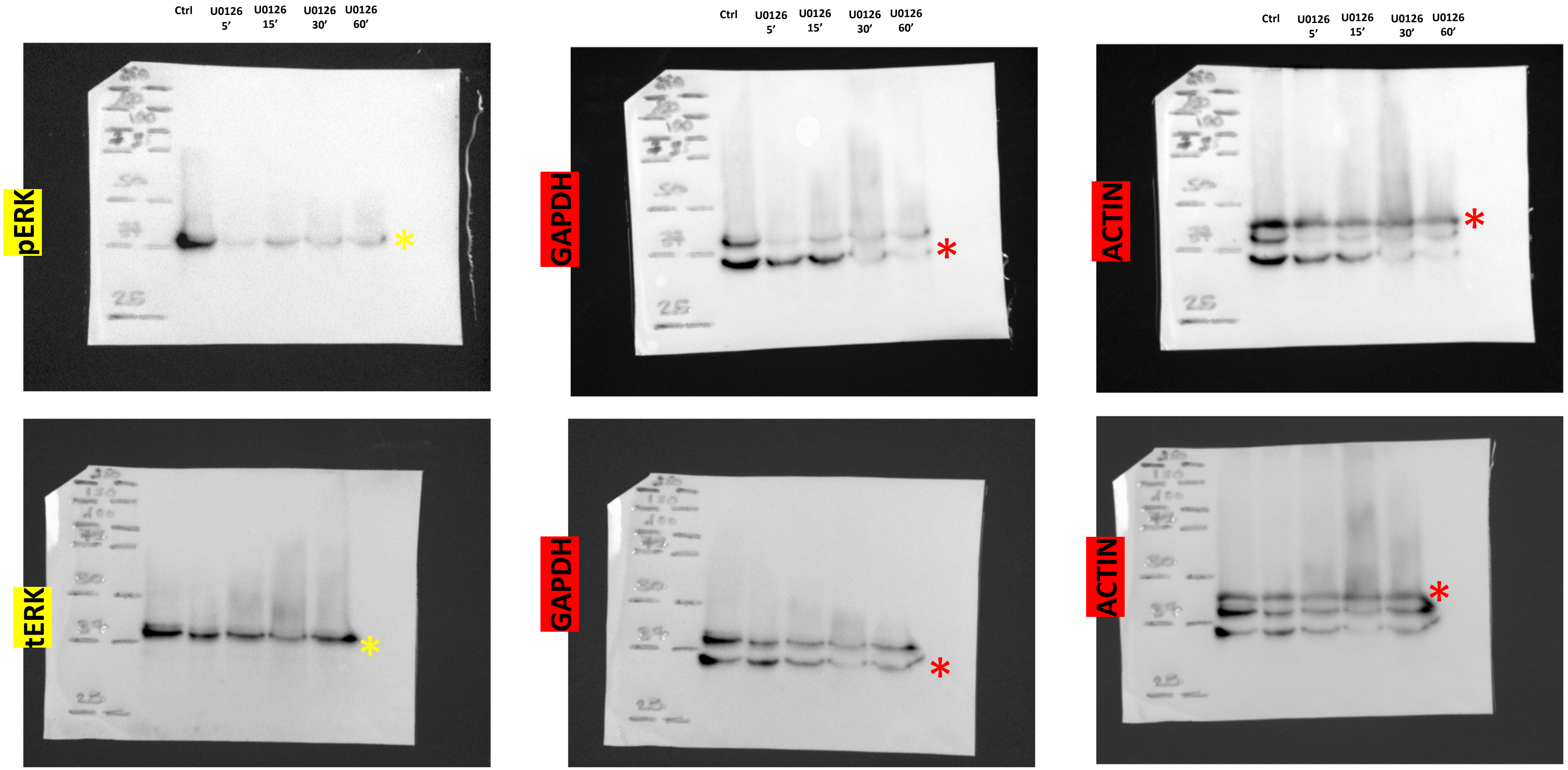

These blots are relative to results presented in FIGURE 2

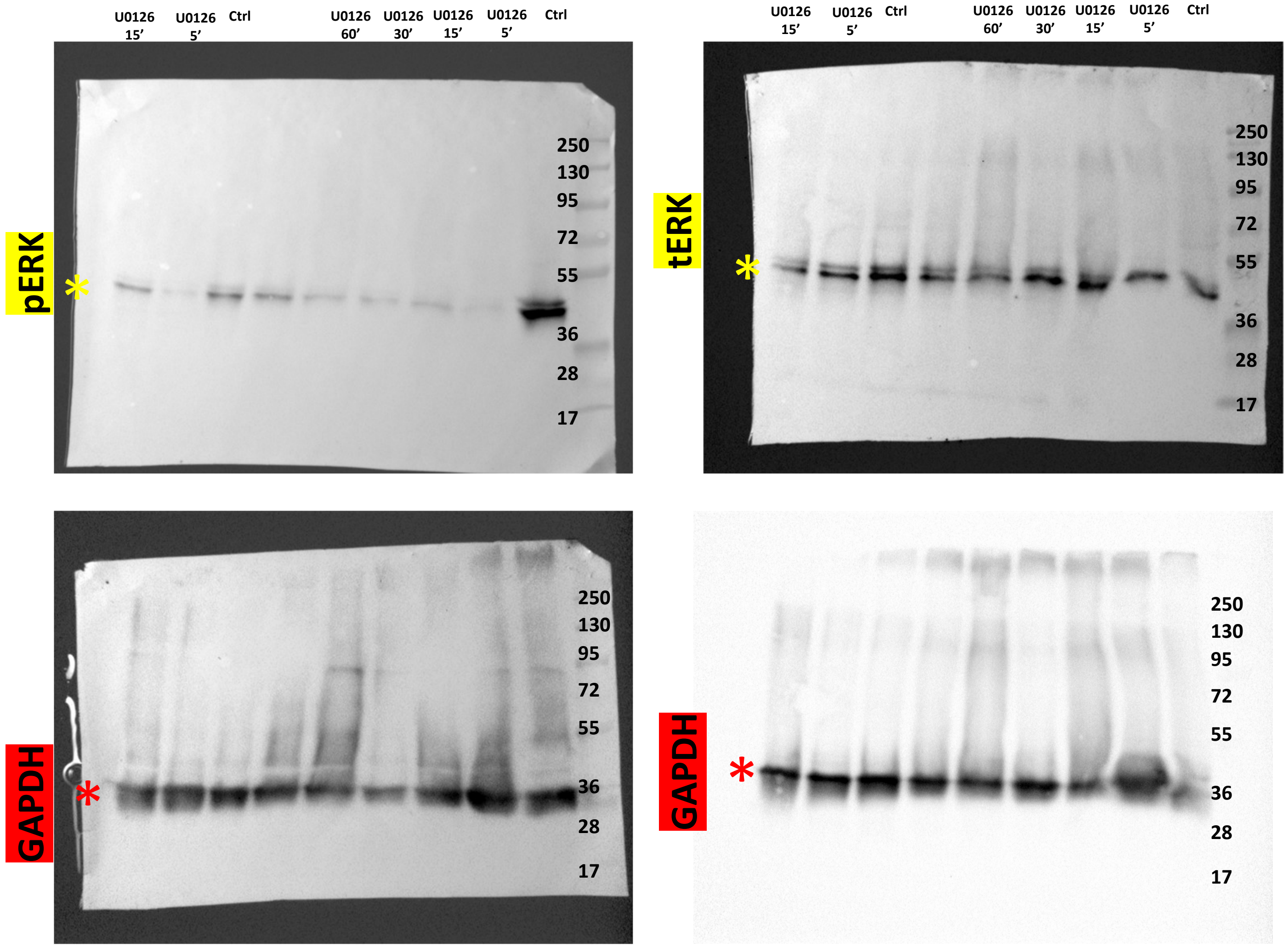

These blots are relative to results presented in FIGURE 2

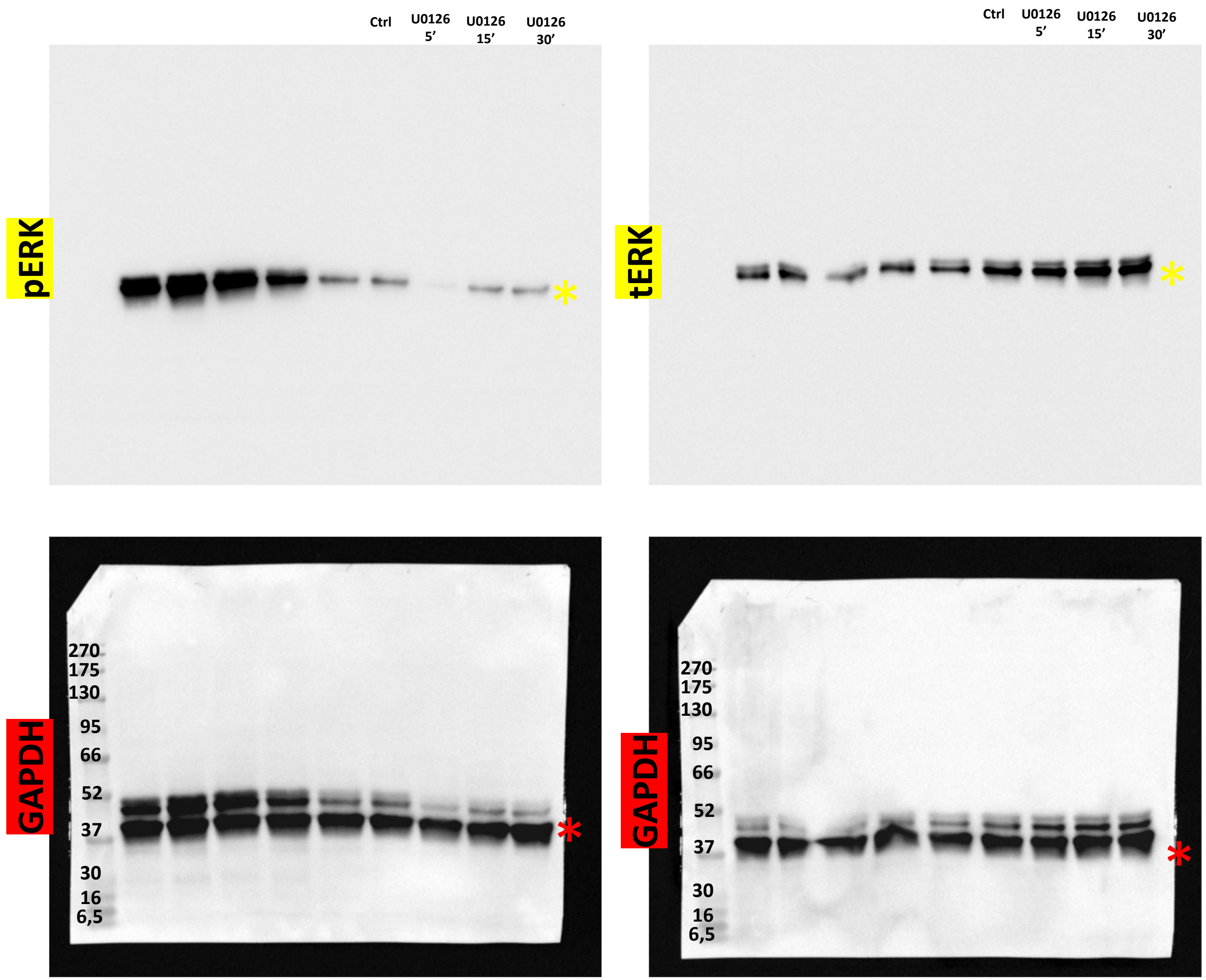

These blots are relative to results presented in FIGURE 2

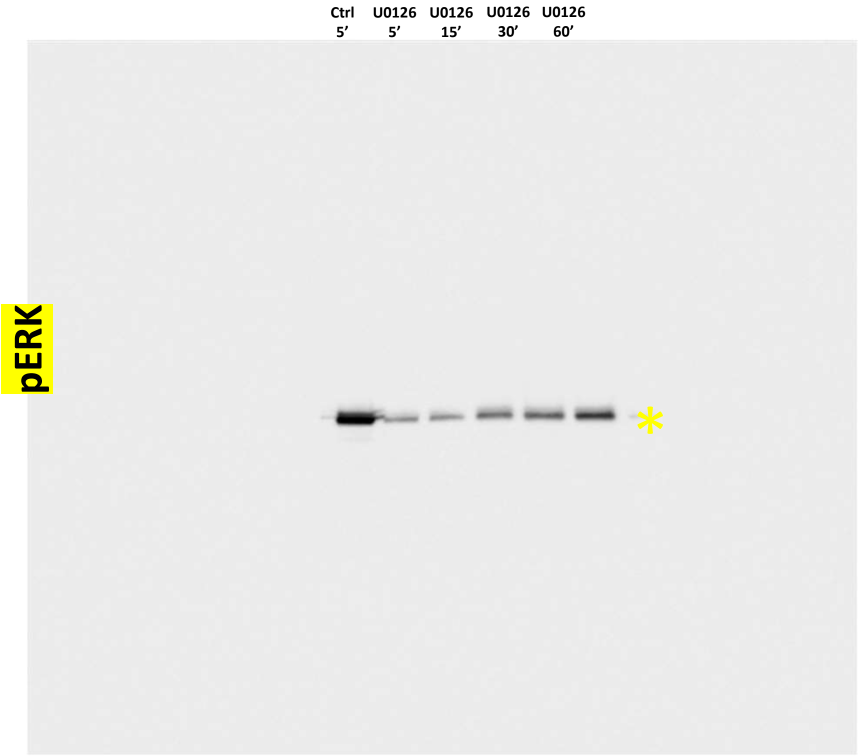

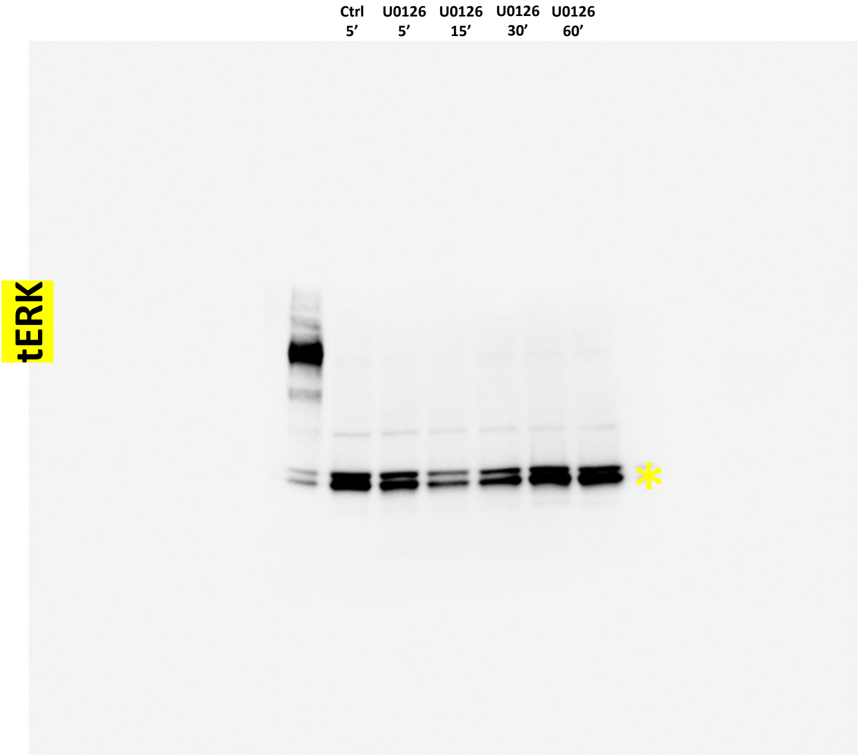

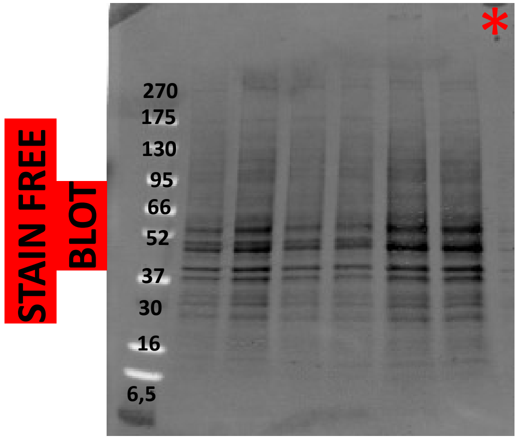

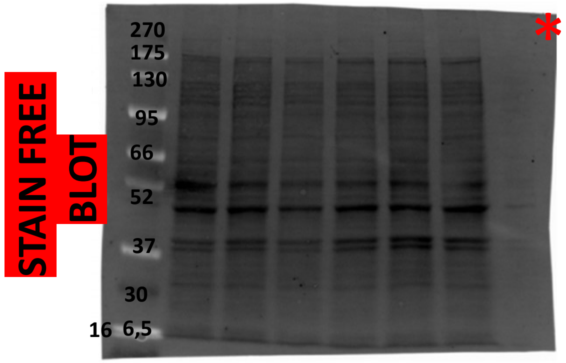

These blots are relative to results presented in FIGURE 2

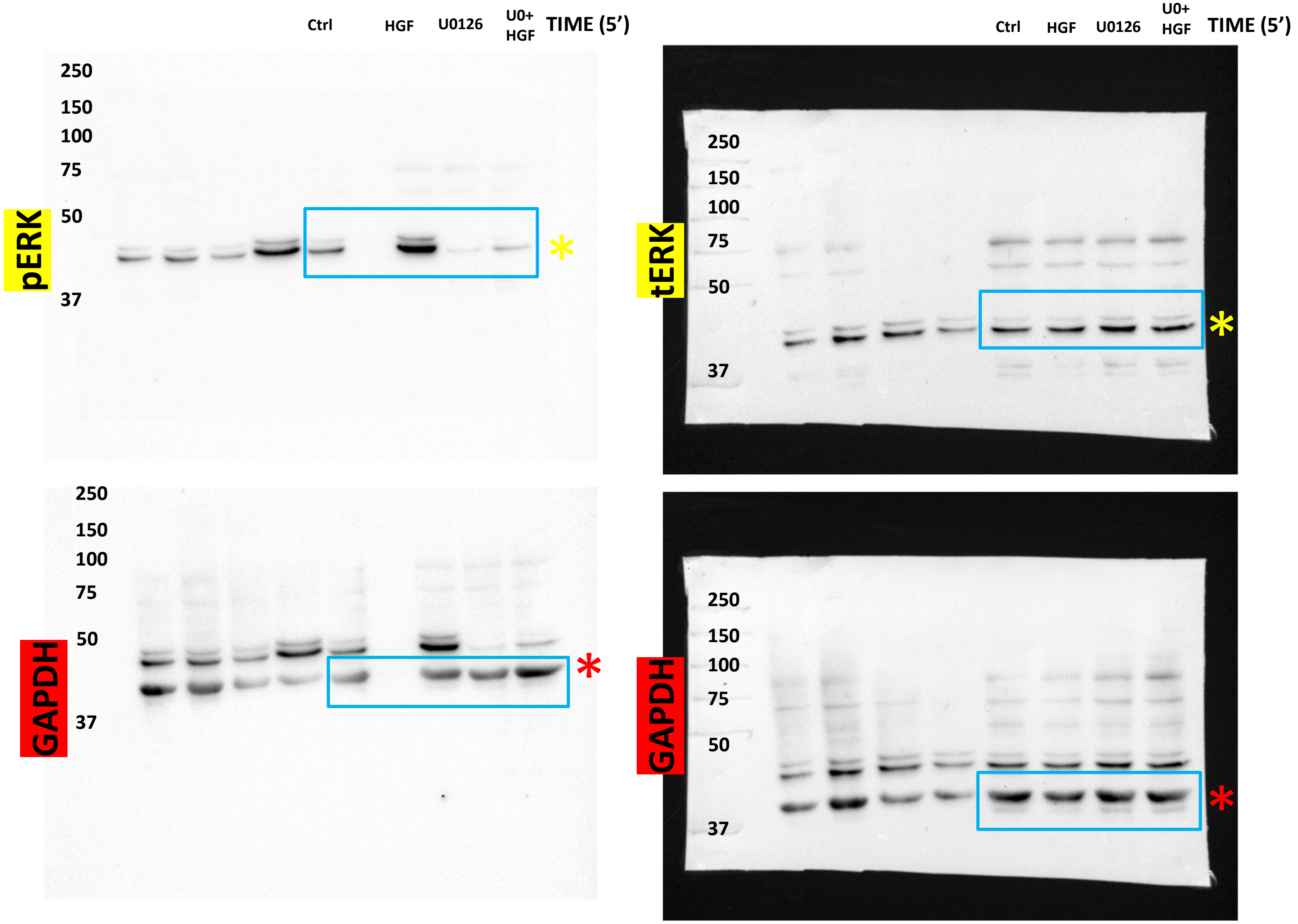

Blue boxes indicate WB lines selected for figures included in the paper.
FIGURE 3A

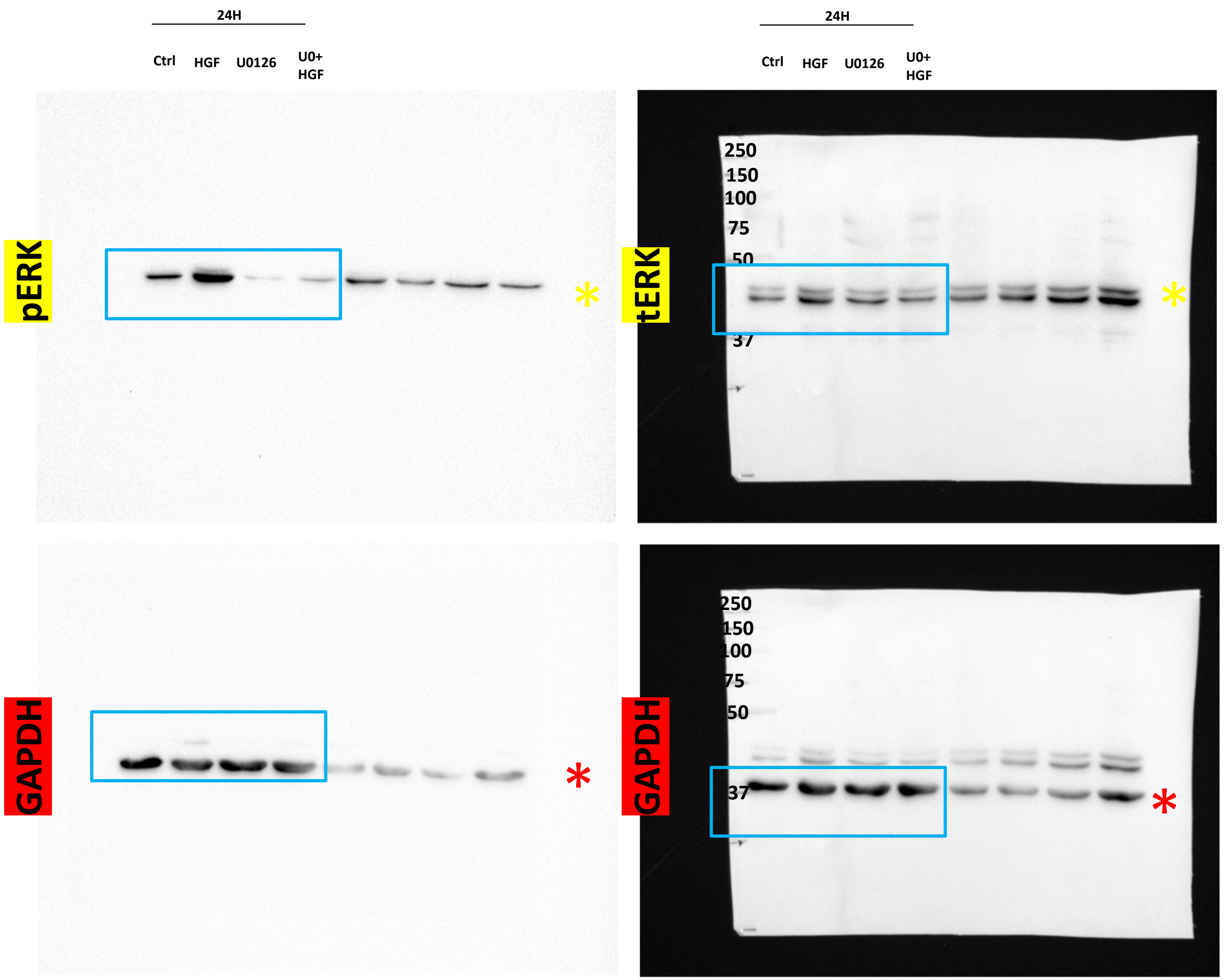

Blue boxes indicate WB lines selected for figures included in the paper. FIGURE 3C

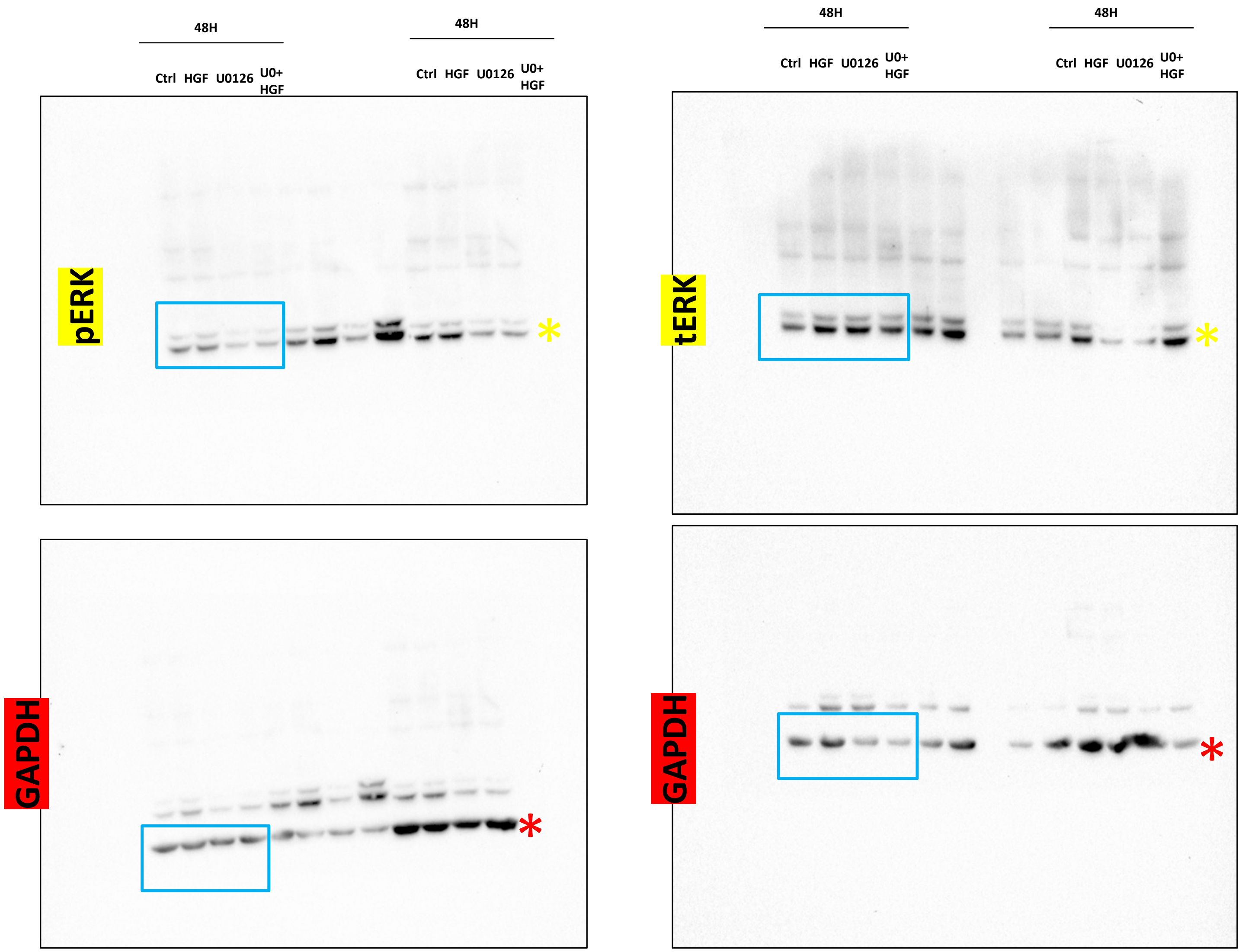

Blue boxes indicate WB lines selected for figures included in the paper.
FIGURE 3E

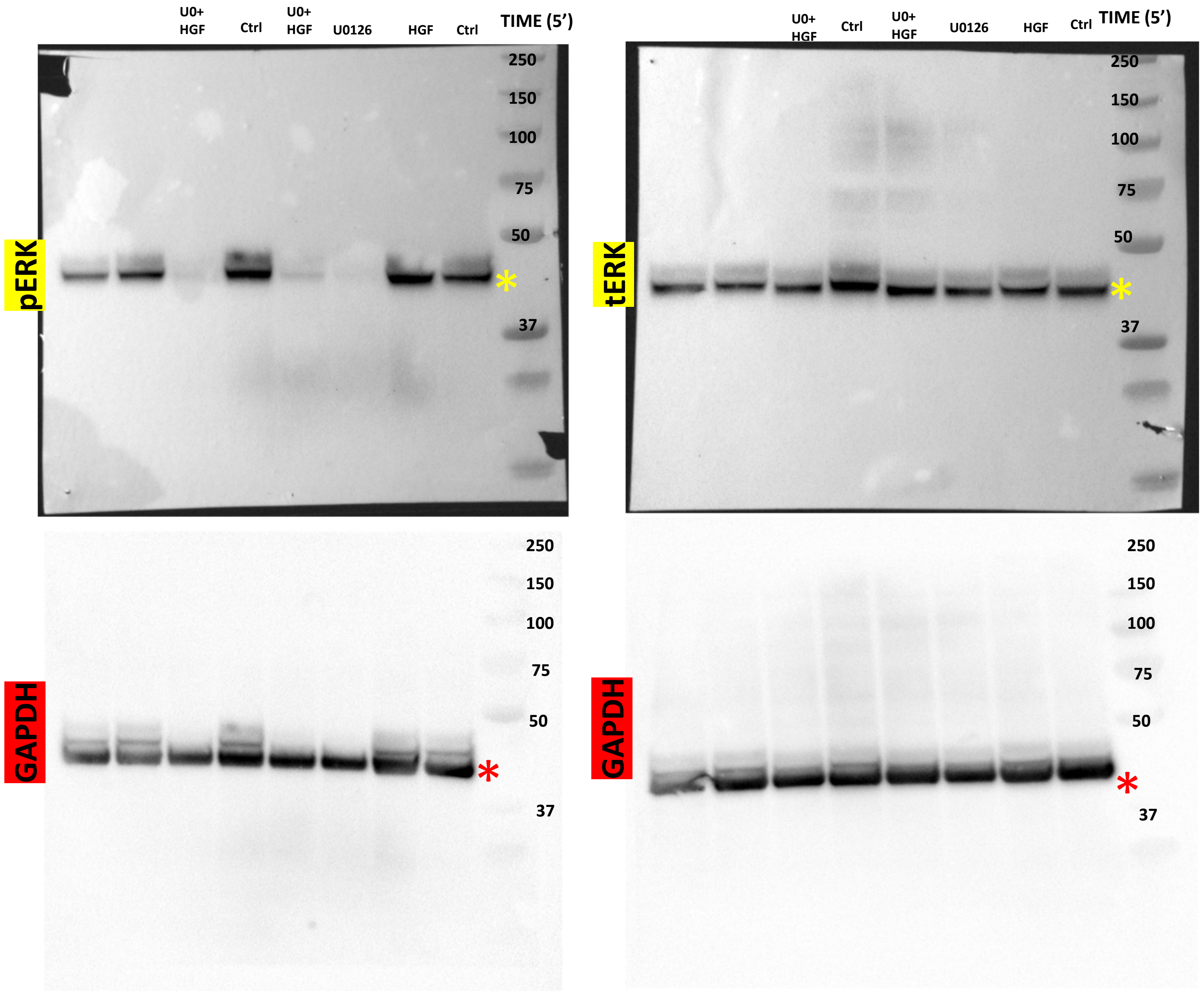

These blots are relative to results presented in FIGURE 3

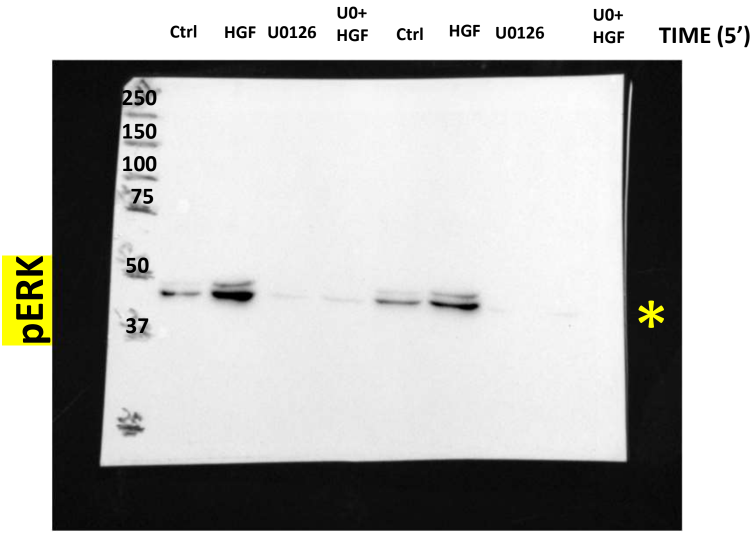

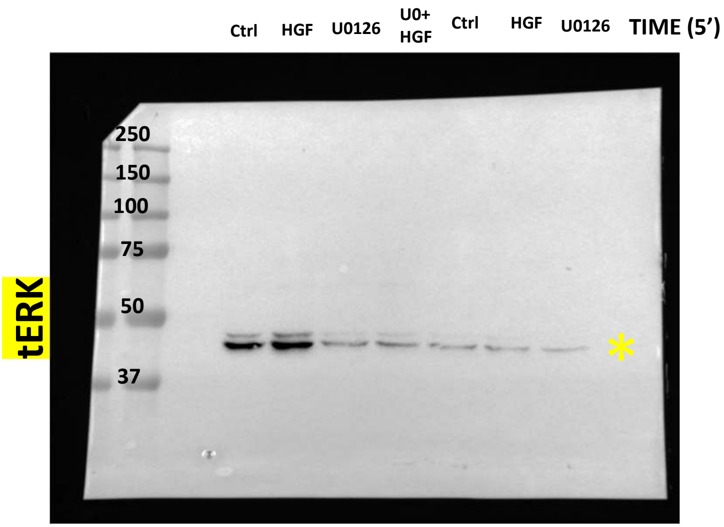

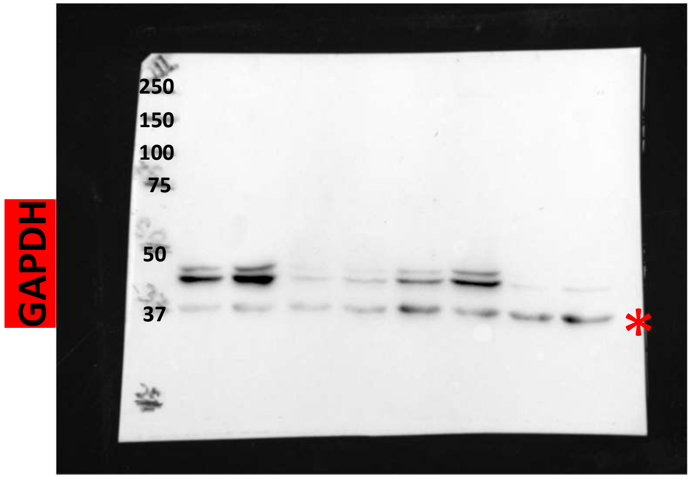

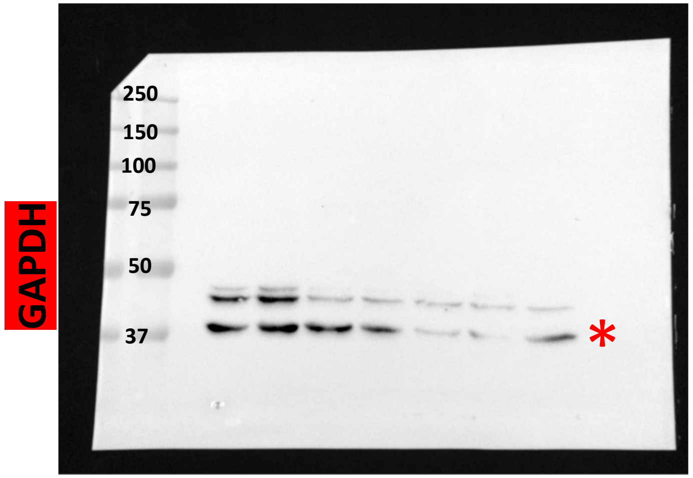

These blots are relative to results presented in FIGURE 3

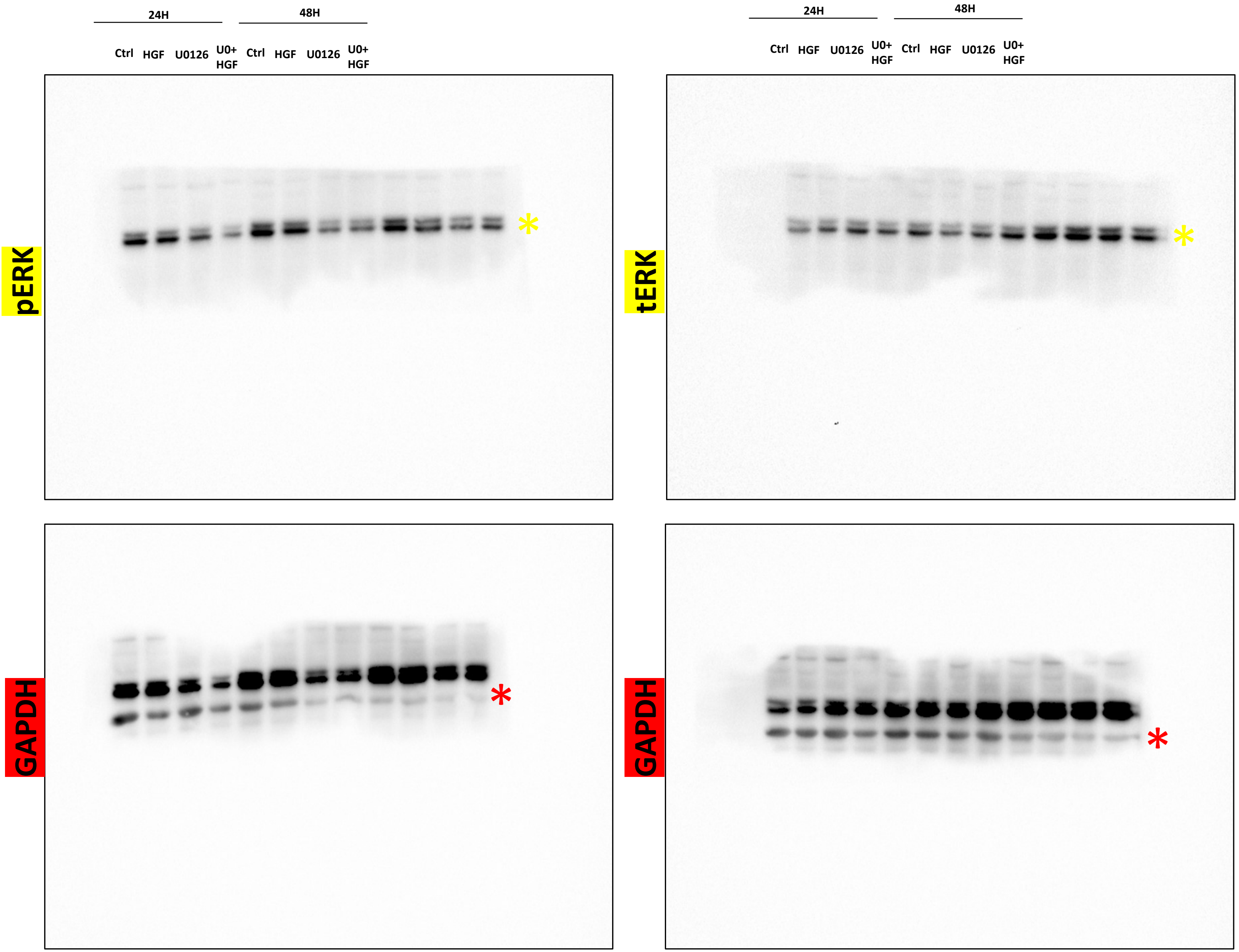

These blots are relative to results presented in FIGURE 3

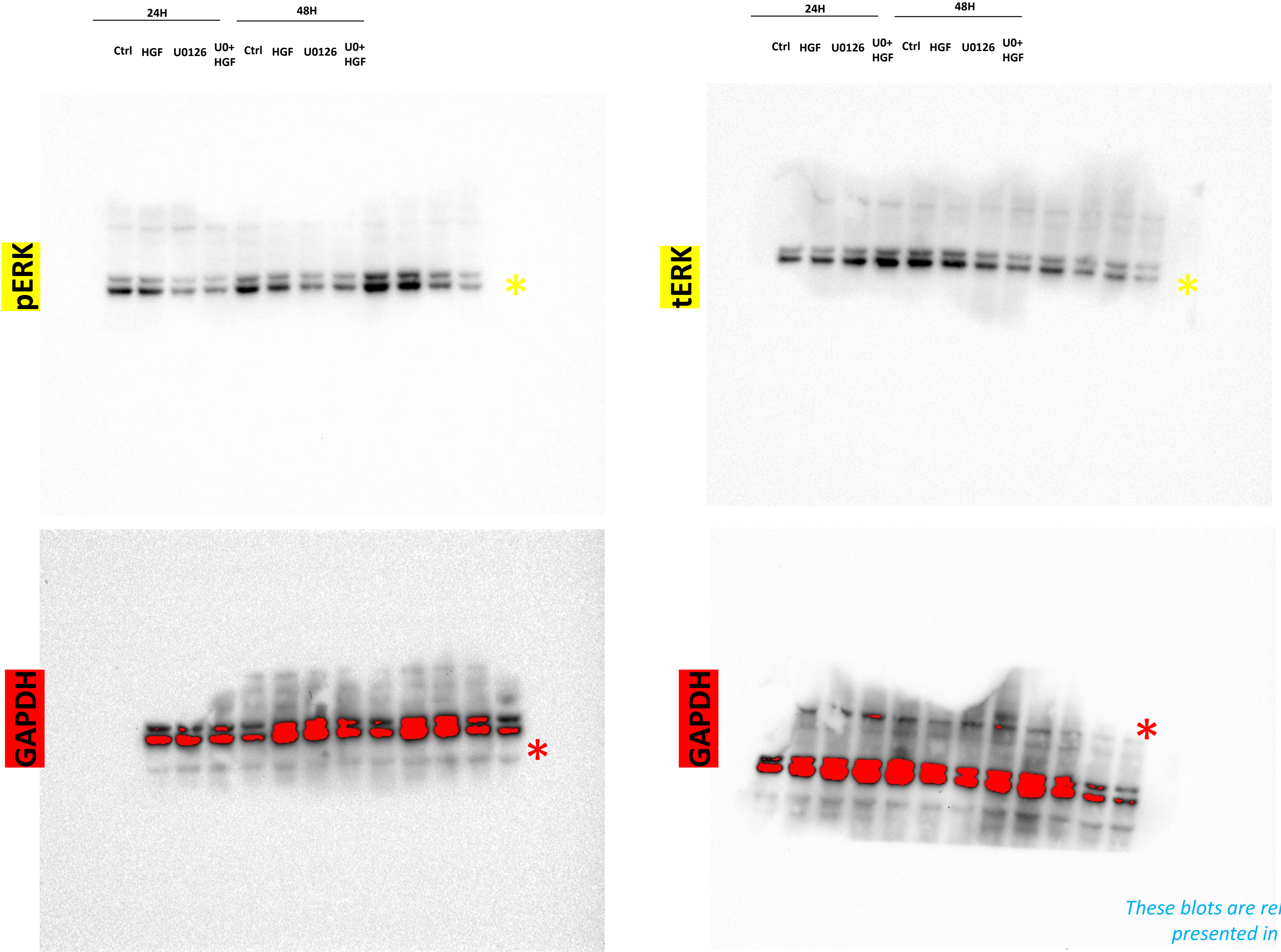

These blots are relative to results presented in FIGURE 3

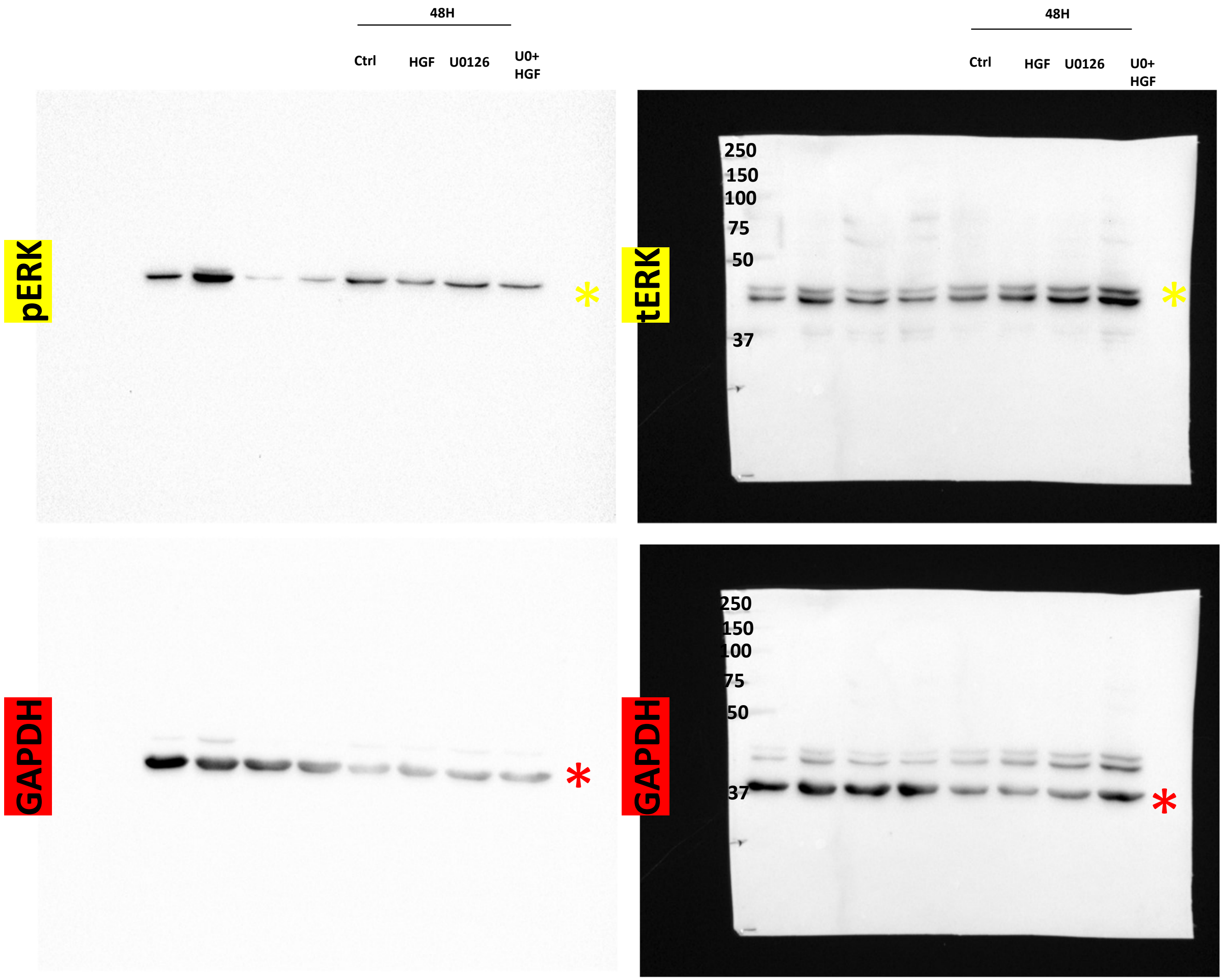

These blots are relative to results presented in FIGURE 3

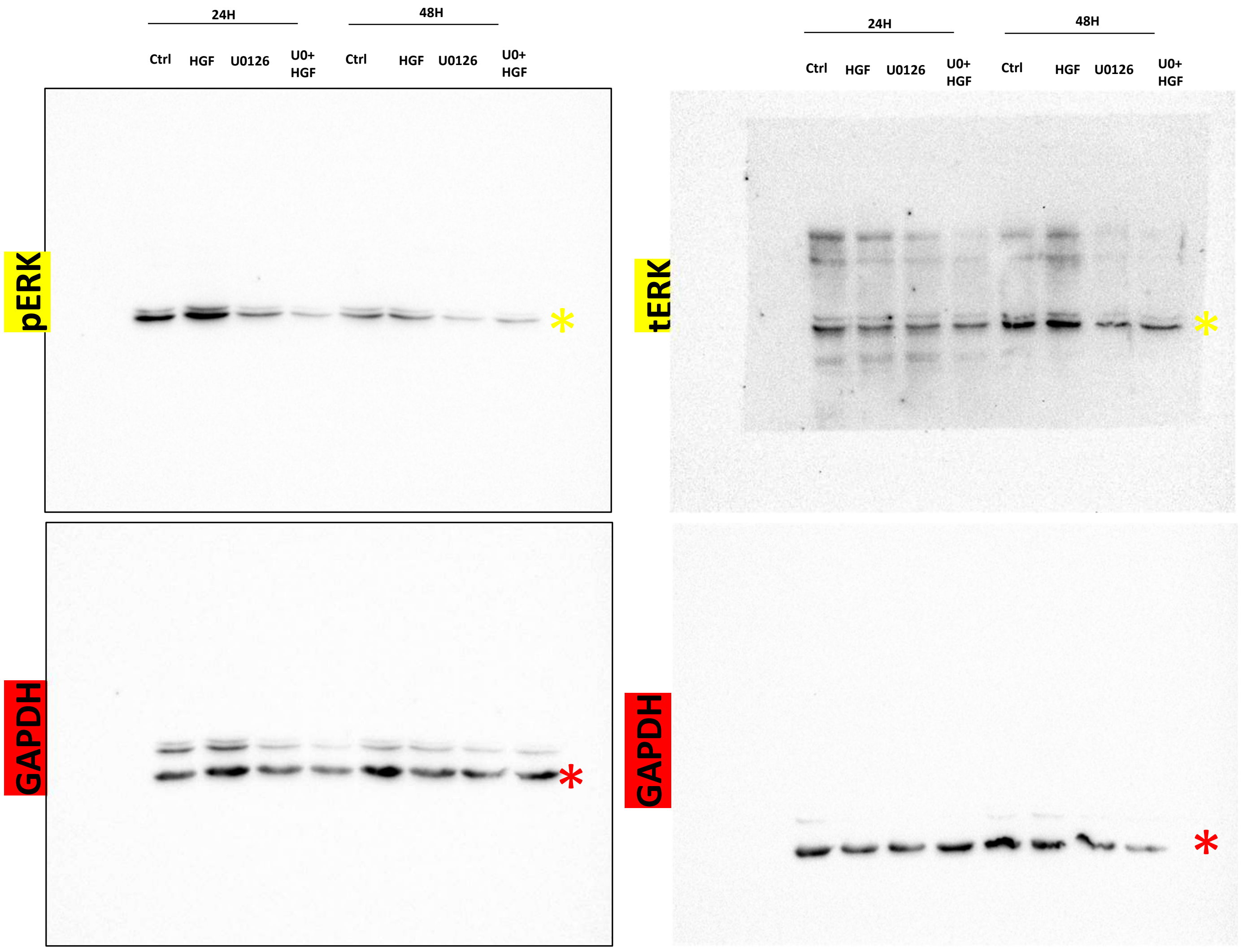

These blots are relative to results presented in FIGURE 3

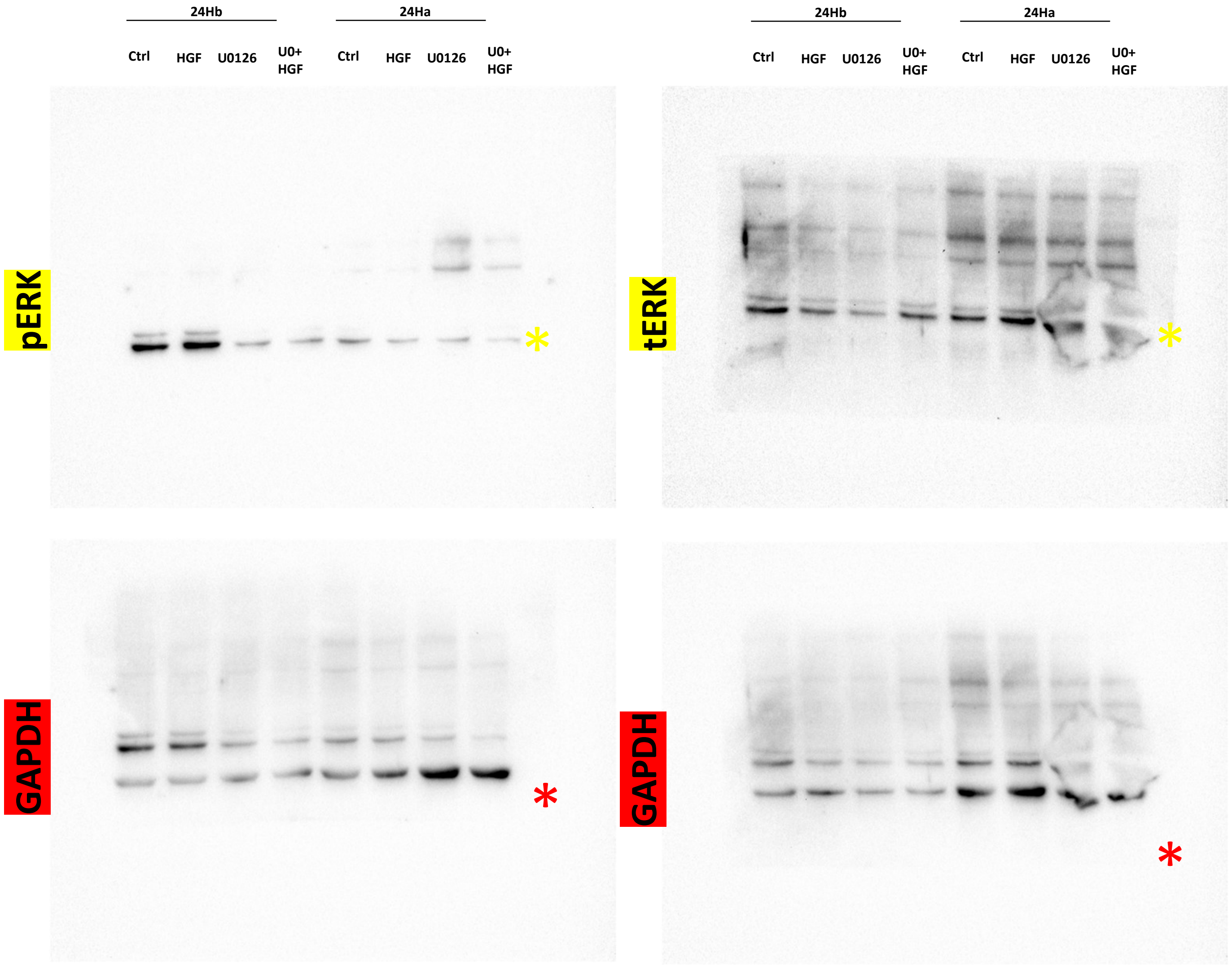

These blots are relative to results presented in FIGURE 3

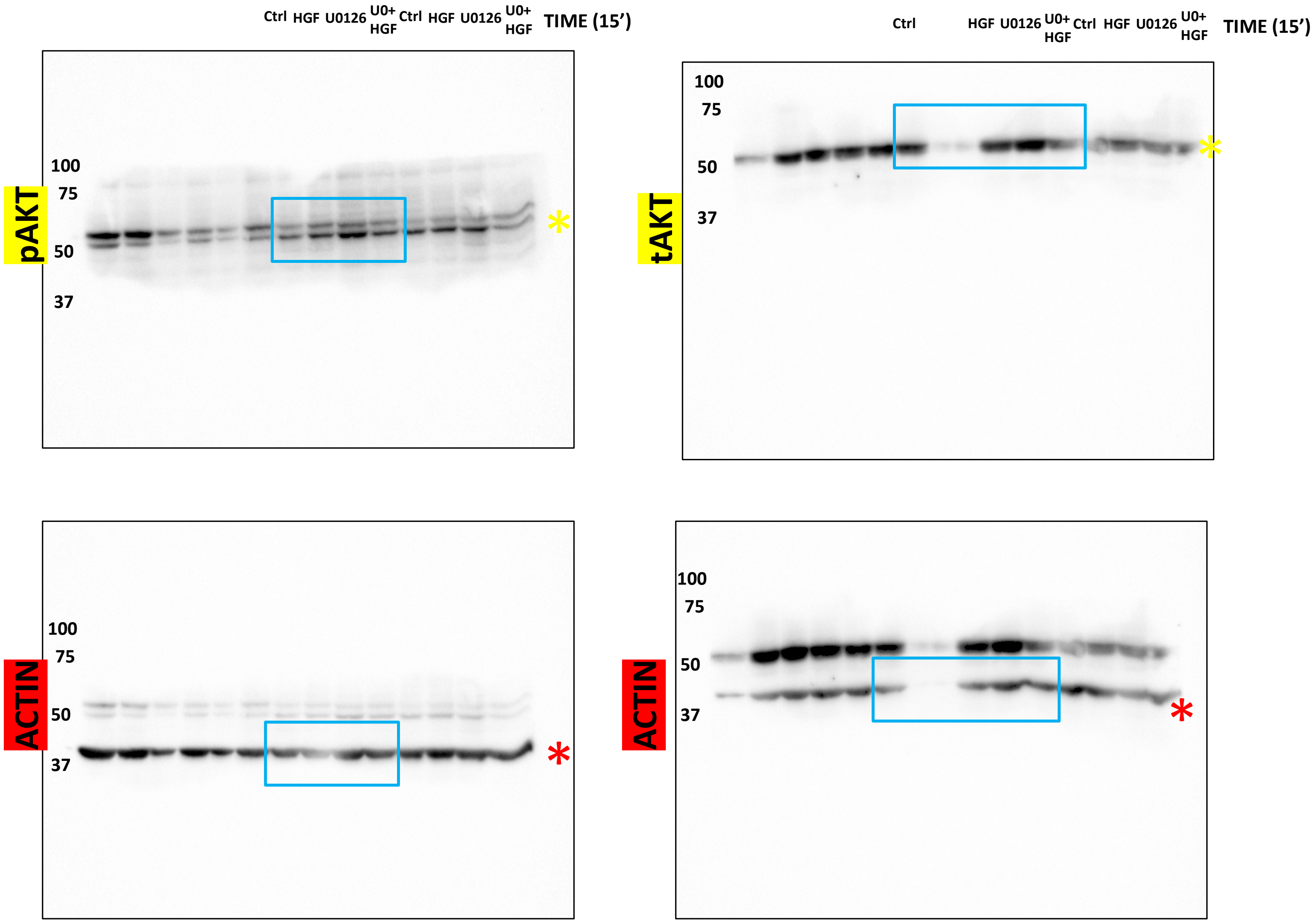

Blue boxes indicate WB lines selected for figures included in the paper. FIGURE 7A

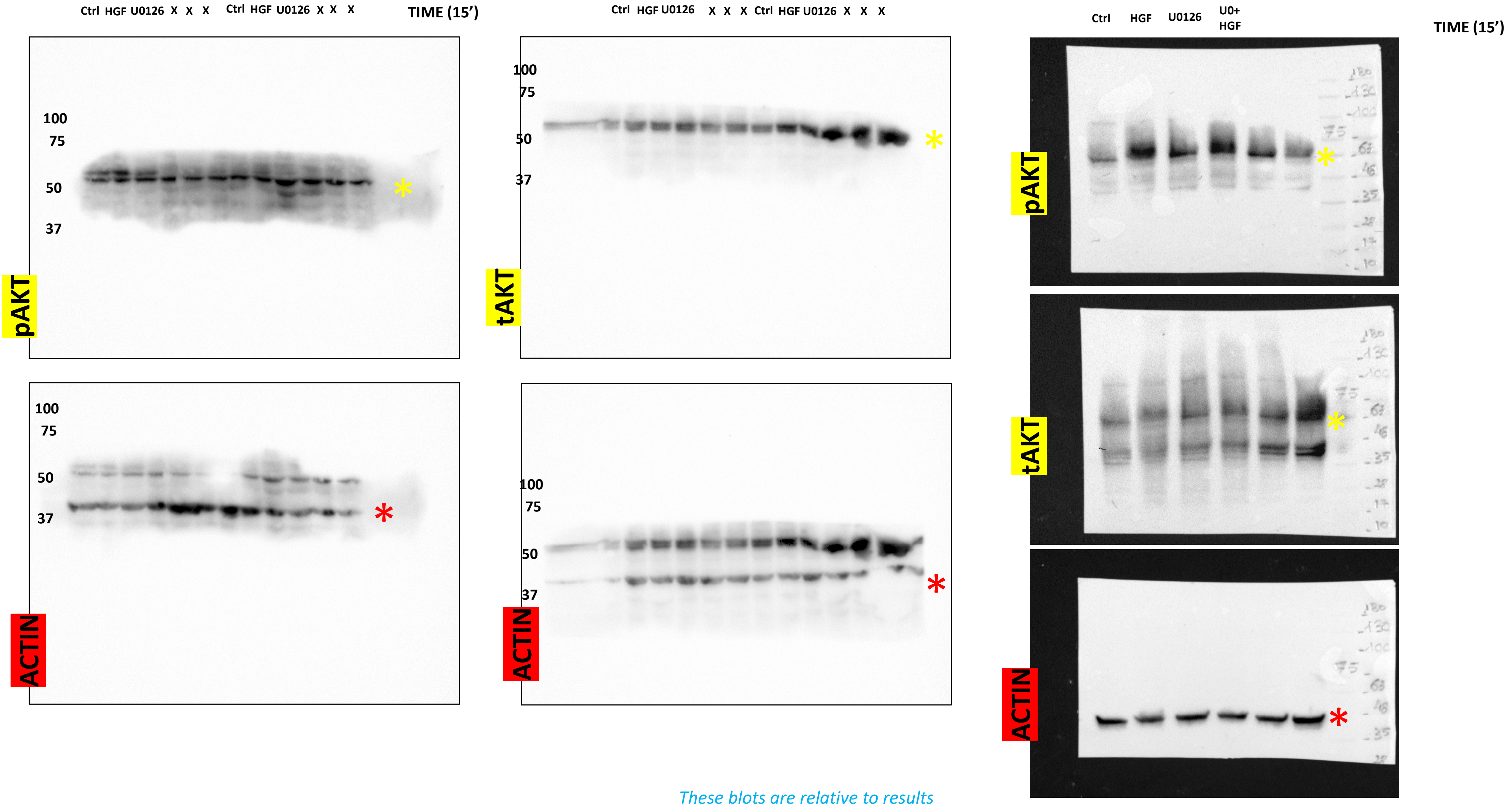

These blots are relative to results presented in FIGURE 7

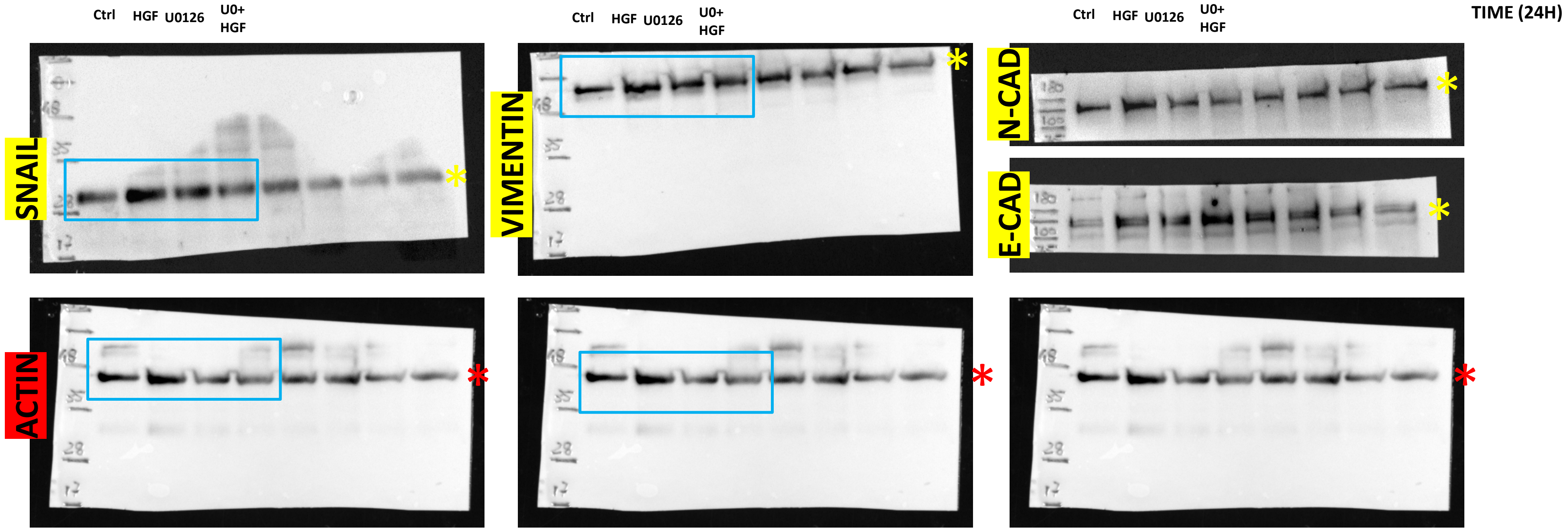

Blue boxes indicate WB lines selected for figures included in the paper. FIGURES 8 A, 8 C

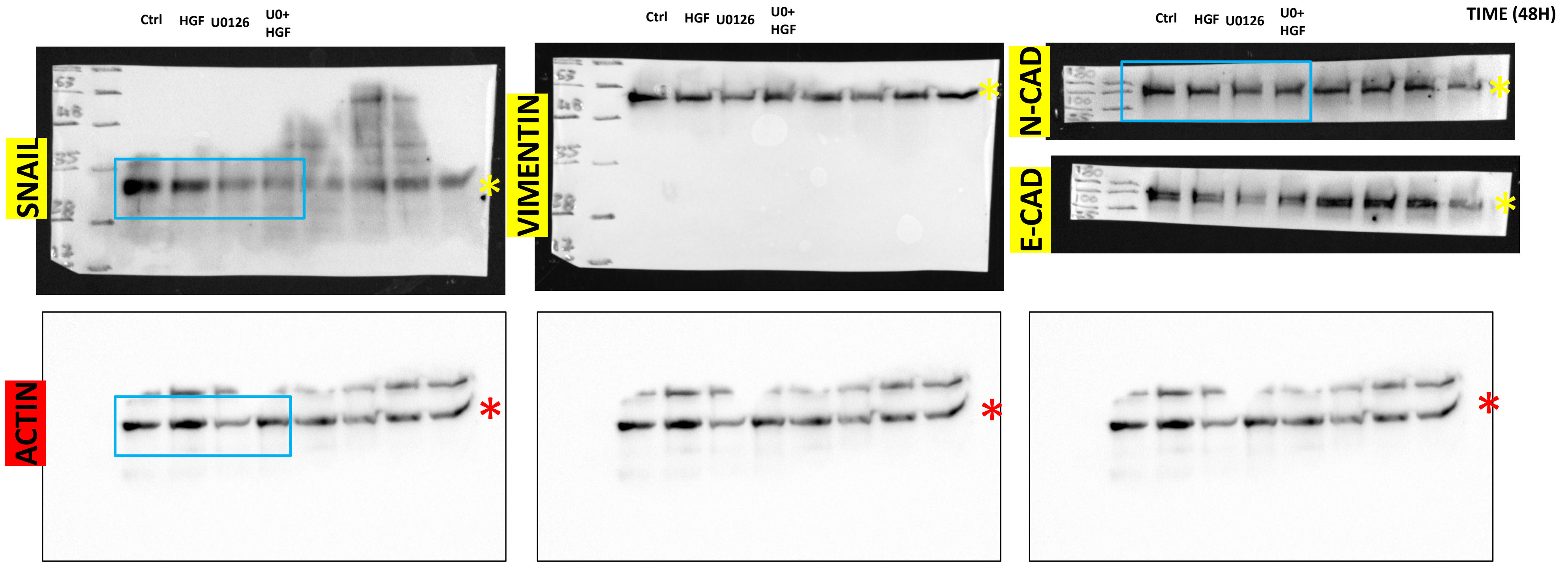

Blue boxes indicate WB lines selected for figures included in the paper. FIGURES 8 B, 8 F

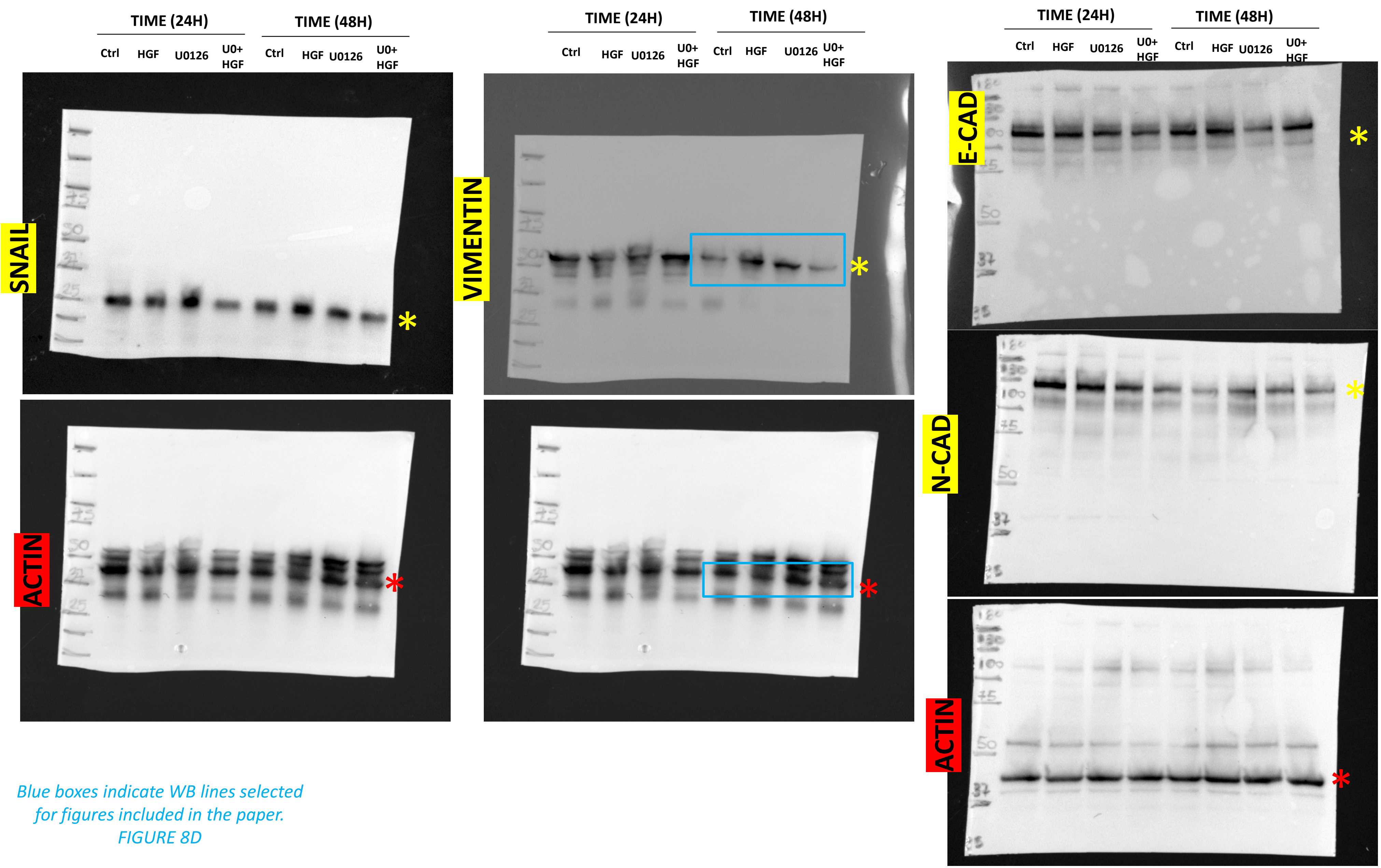

Blue boxes indicate WB lines selected for figures included in the paper.
FIGURE 8D

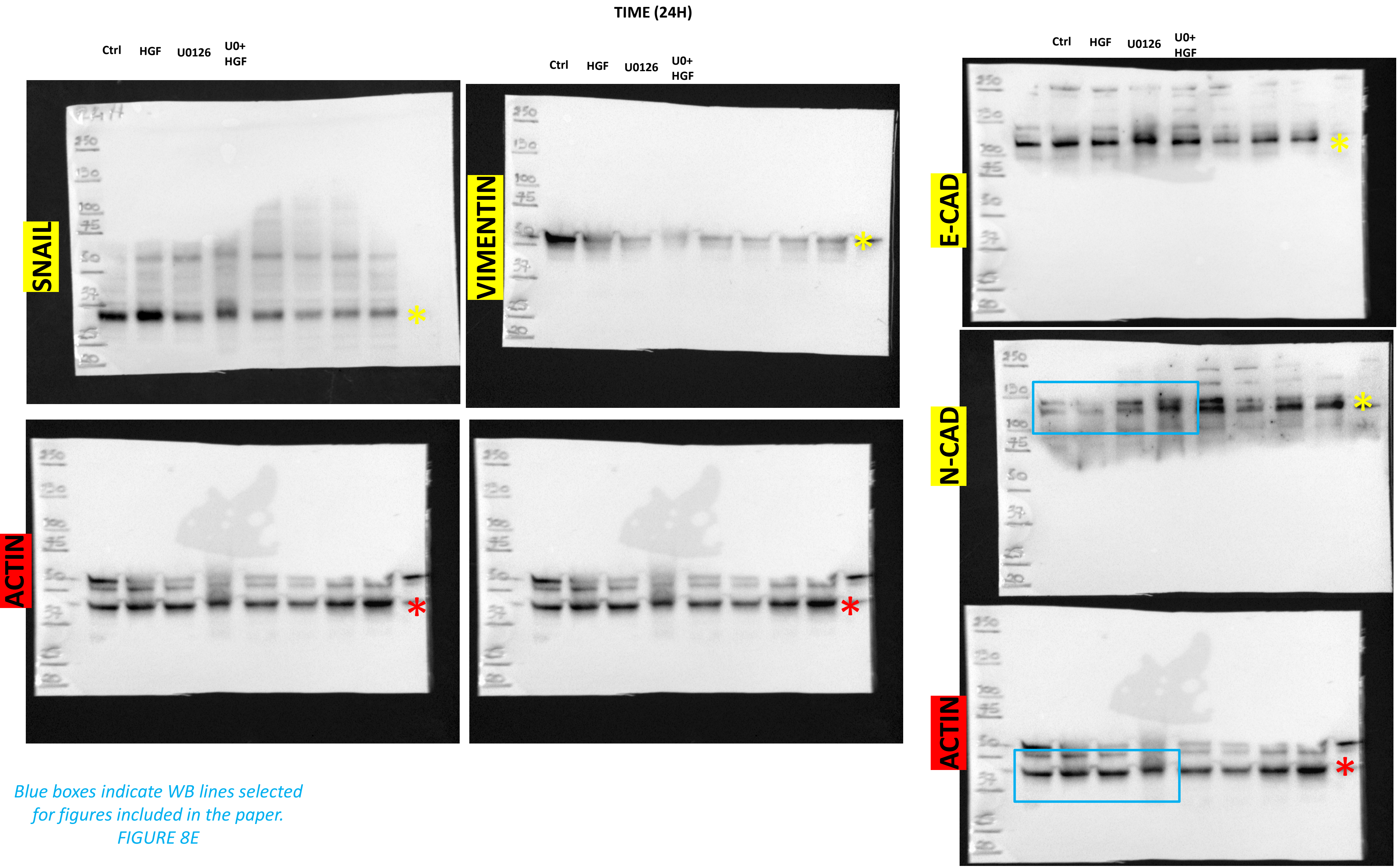

Blue boxes indicate WB lines selected for figures included in the paper.
FIGURE 8E

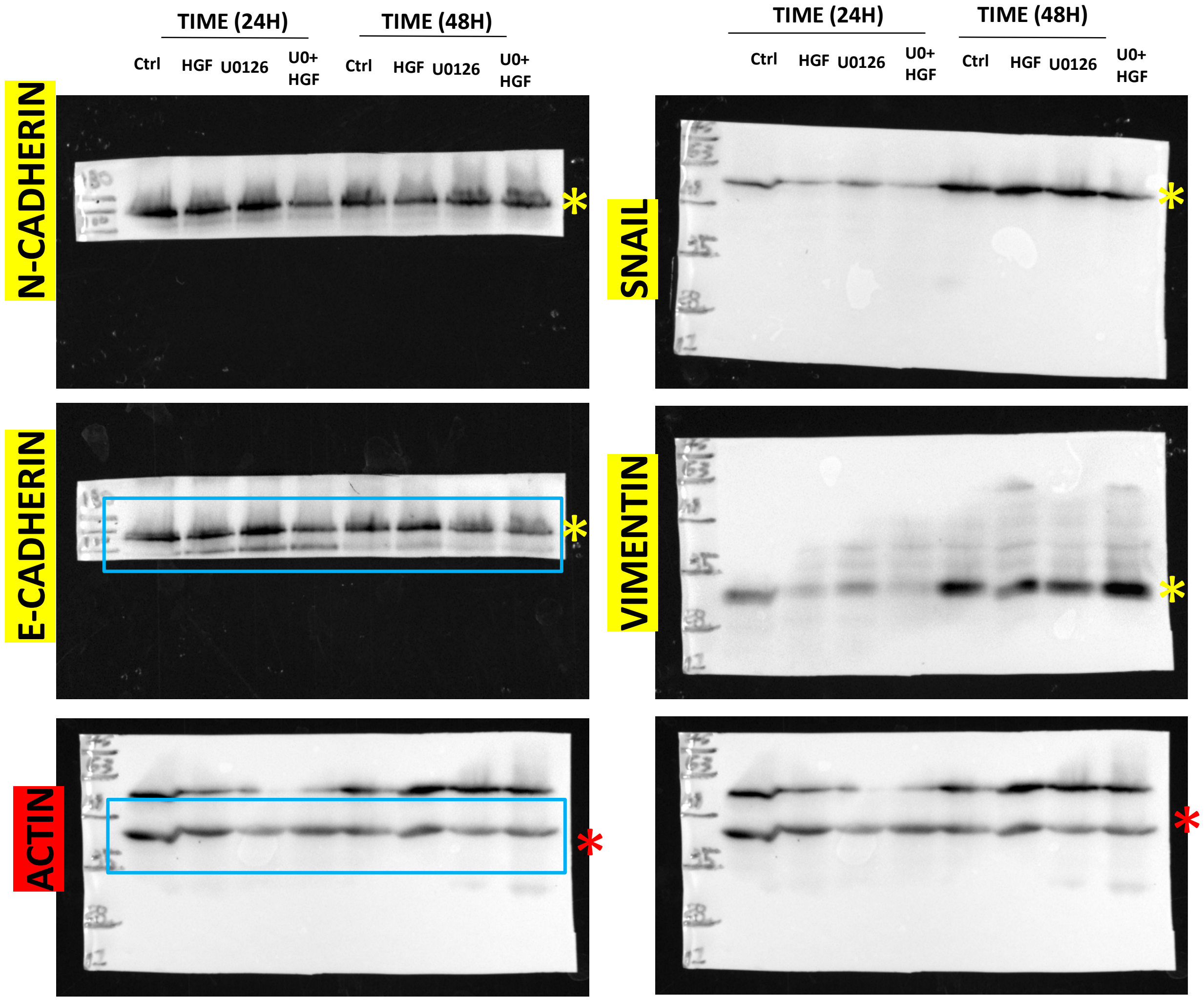

Blue boxes indicate WB lines selected for figures included in the paper. FIGURES 8 G, 8H

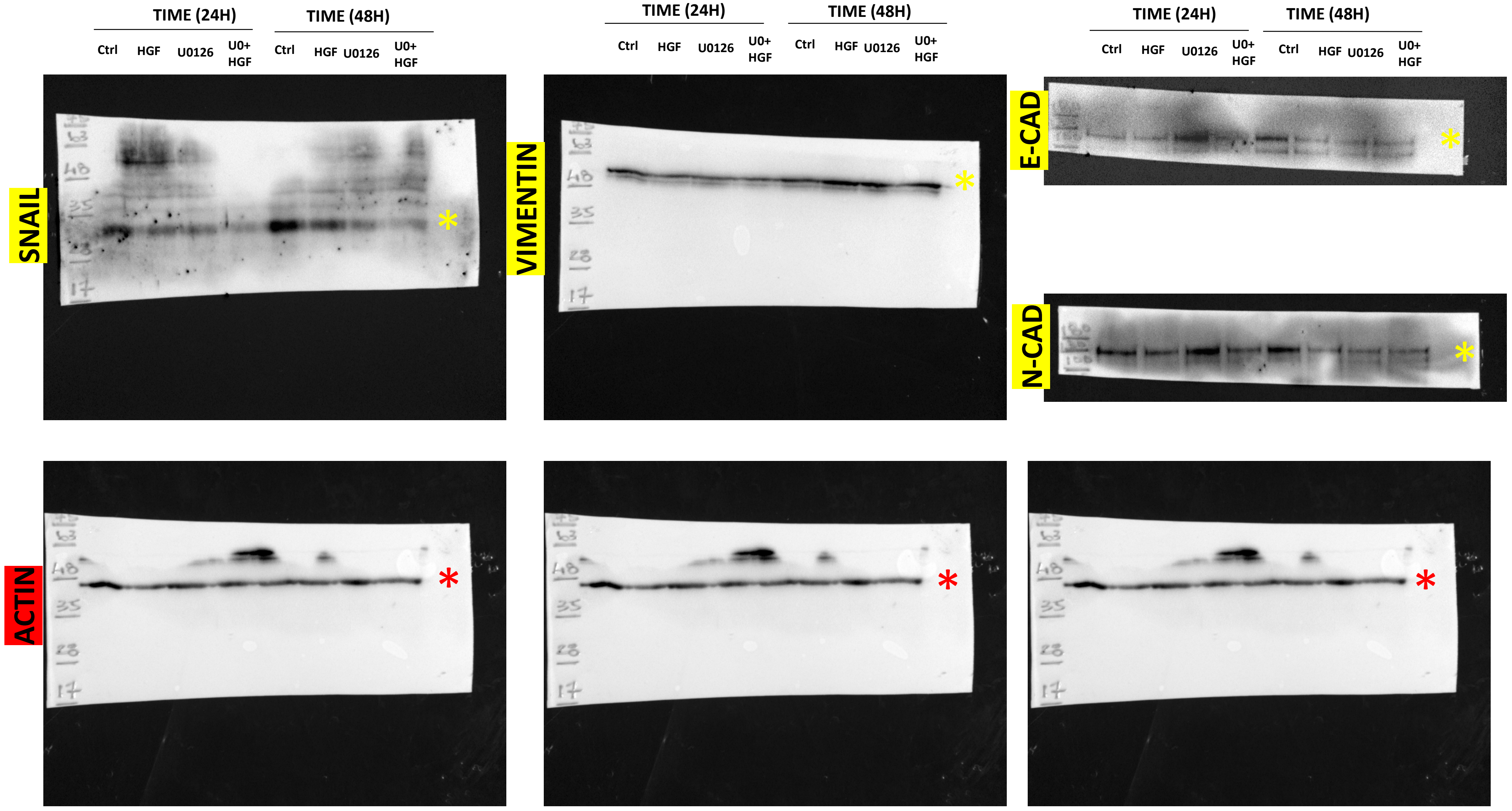

These blots are relative to results presented in FIGURE 8

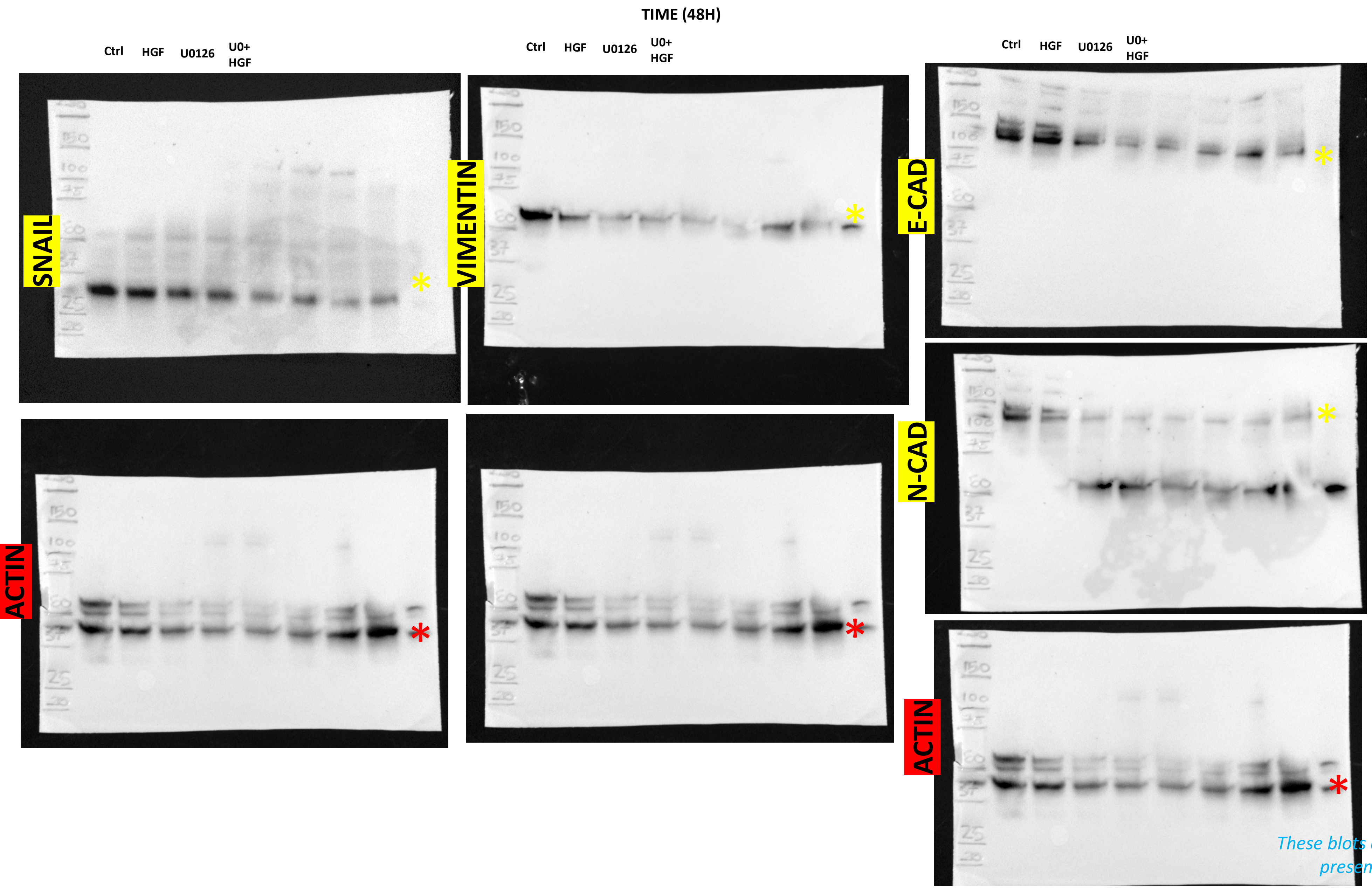

These blots are relative to results presented in FIGURE 8

TIME (48H)

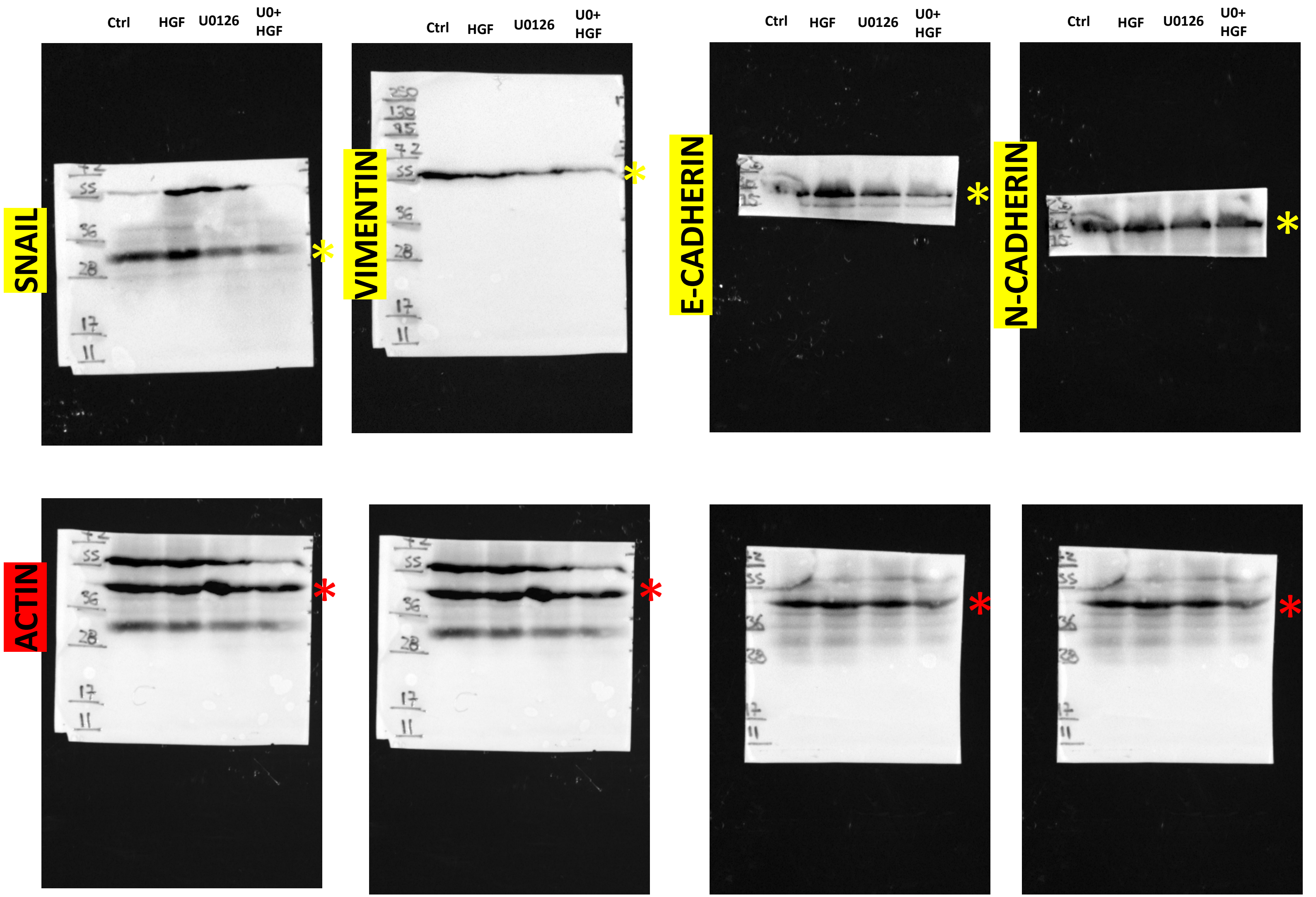

These blots are relative to results presented in FIGURE 8

TIME (24H)

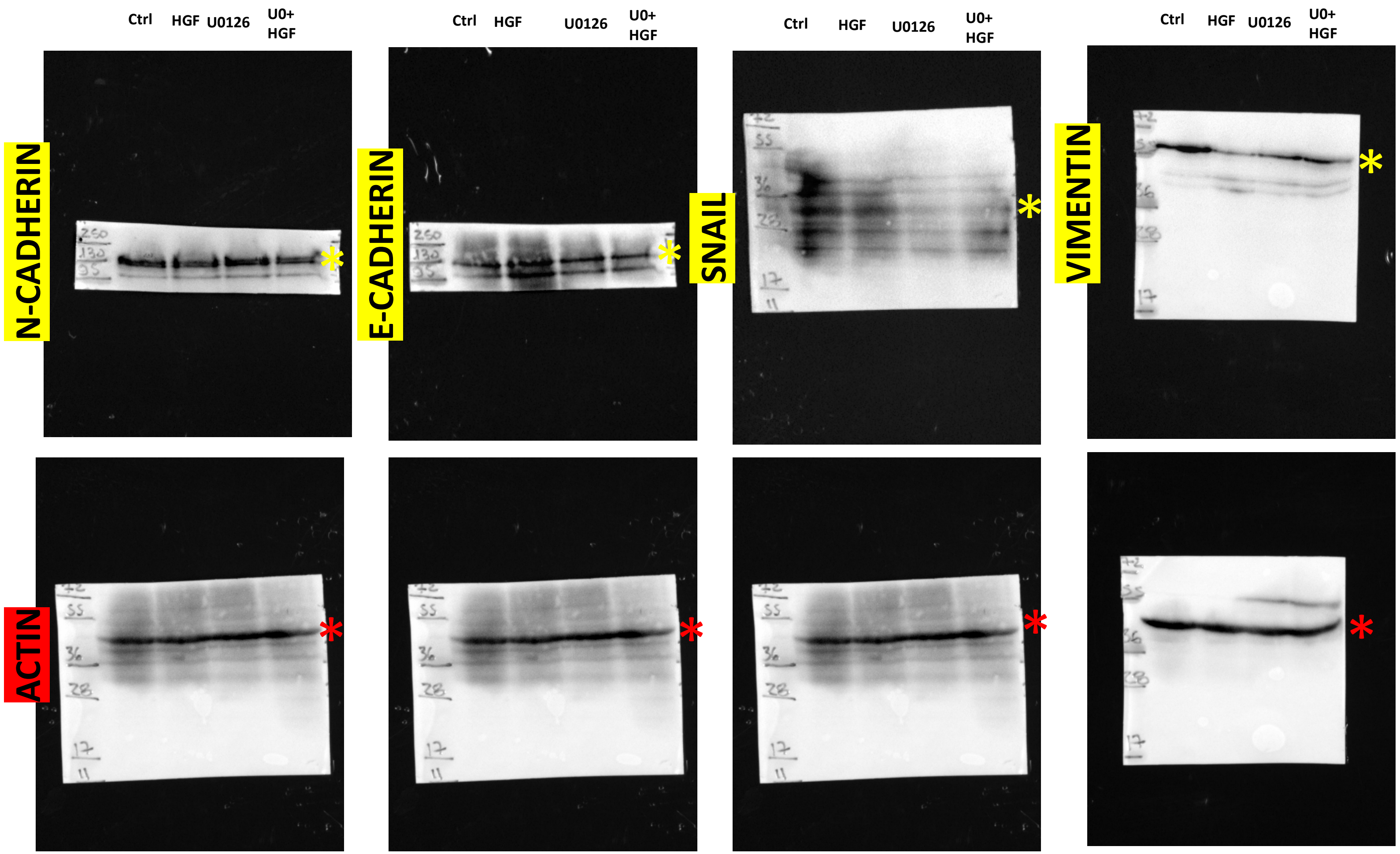

These blots are relative to results presented in FIGURE 8

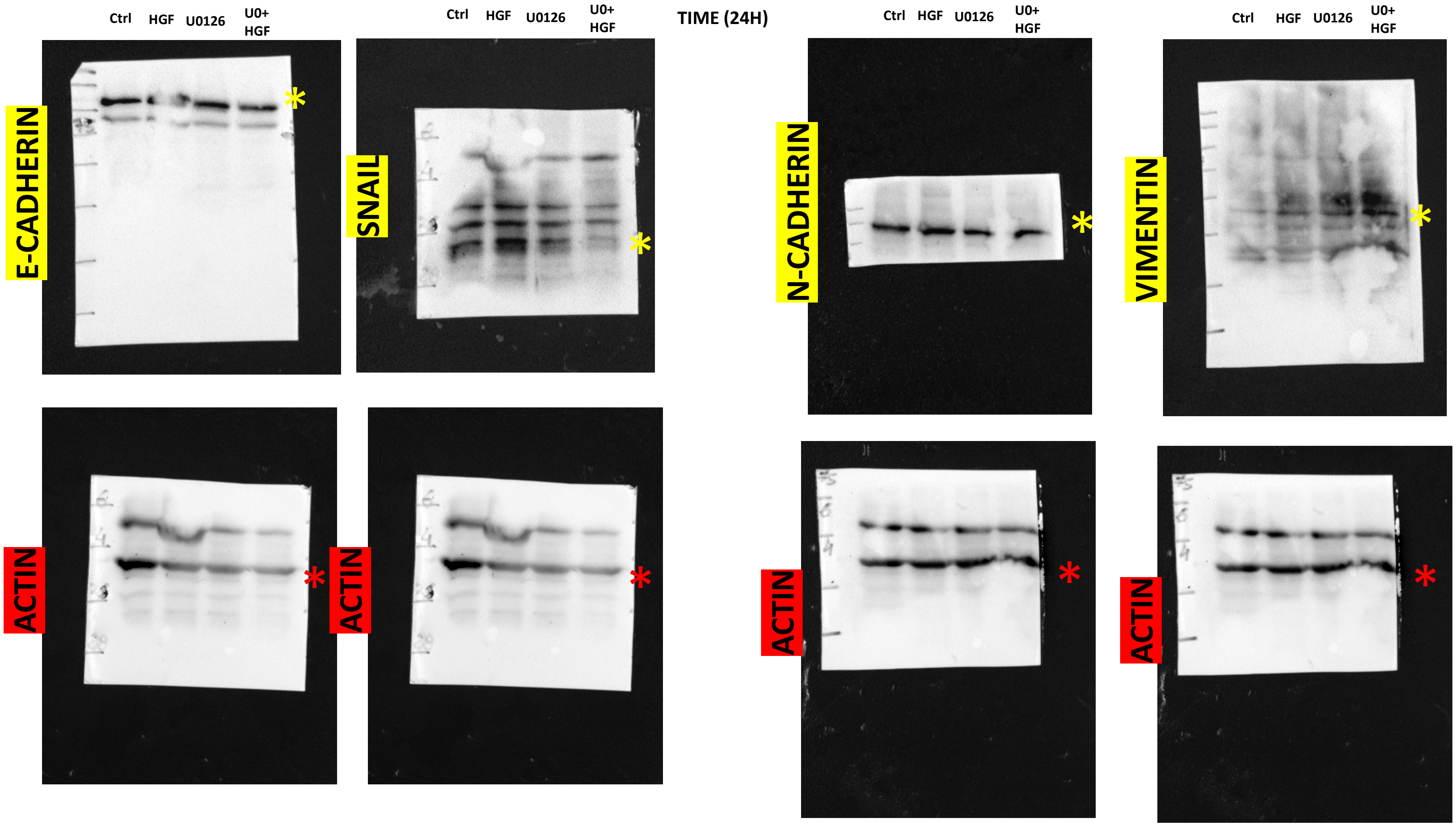

These blots are relative to results presented in FIGURE 8

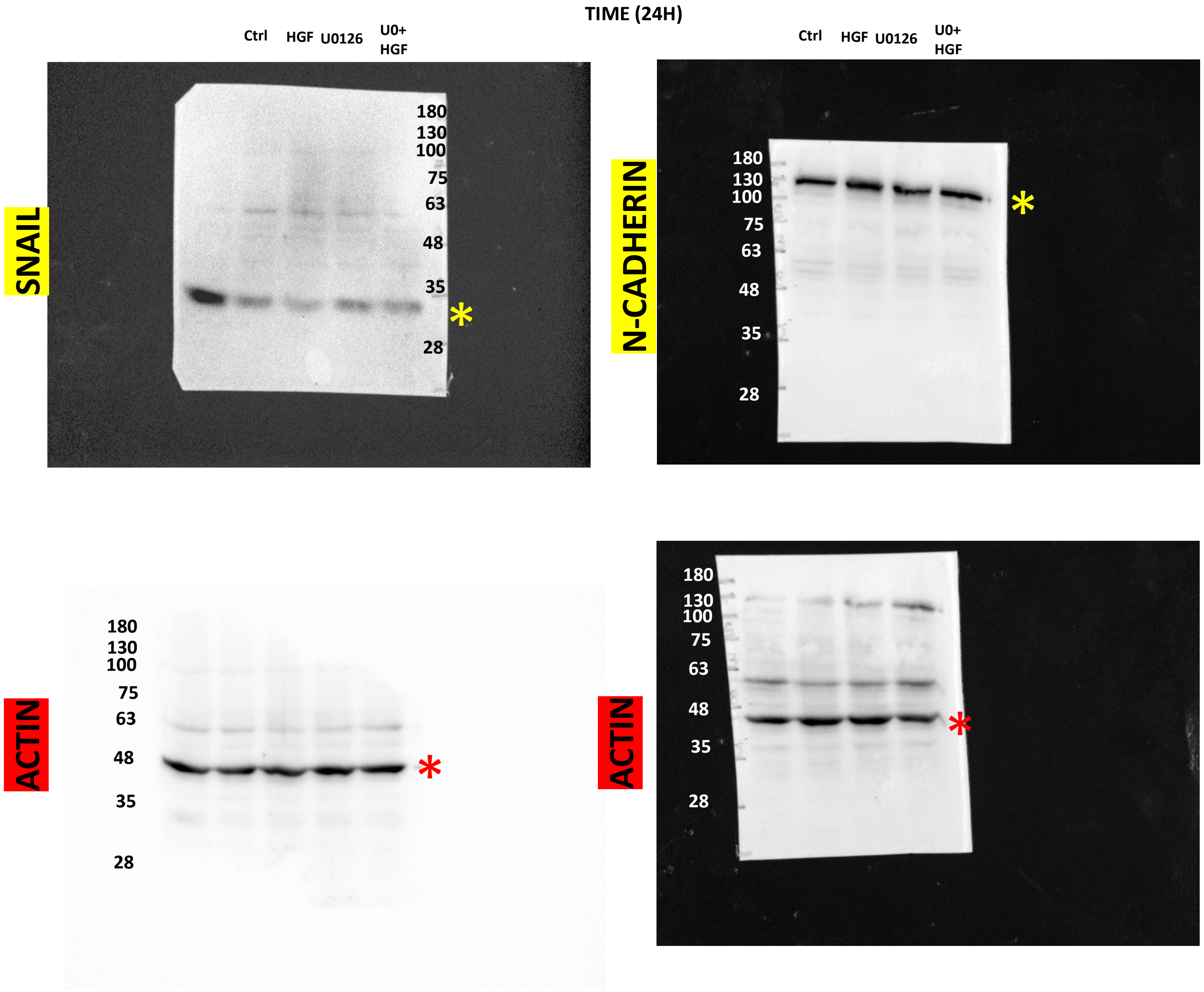

These blots are relative to results presented in FIGURE 8

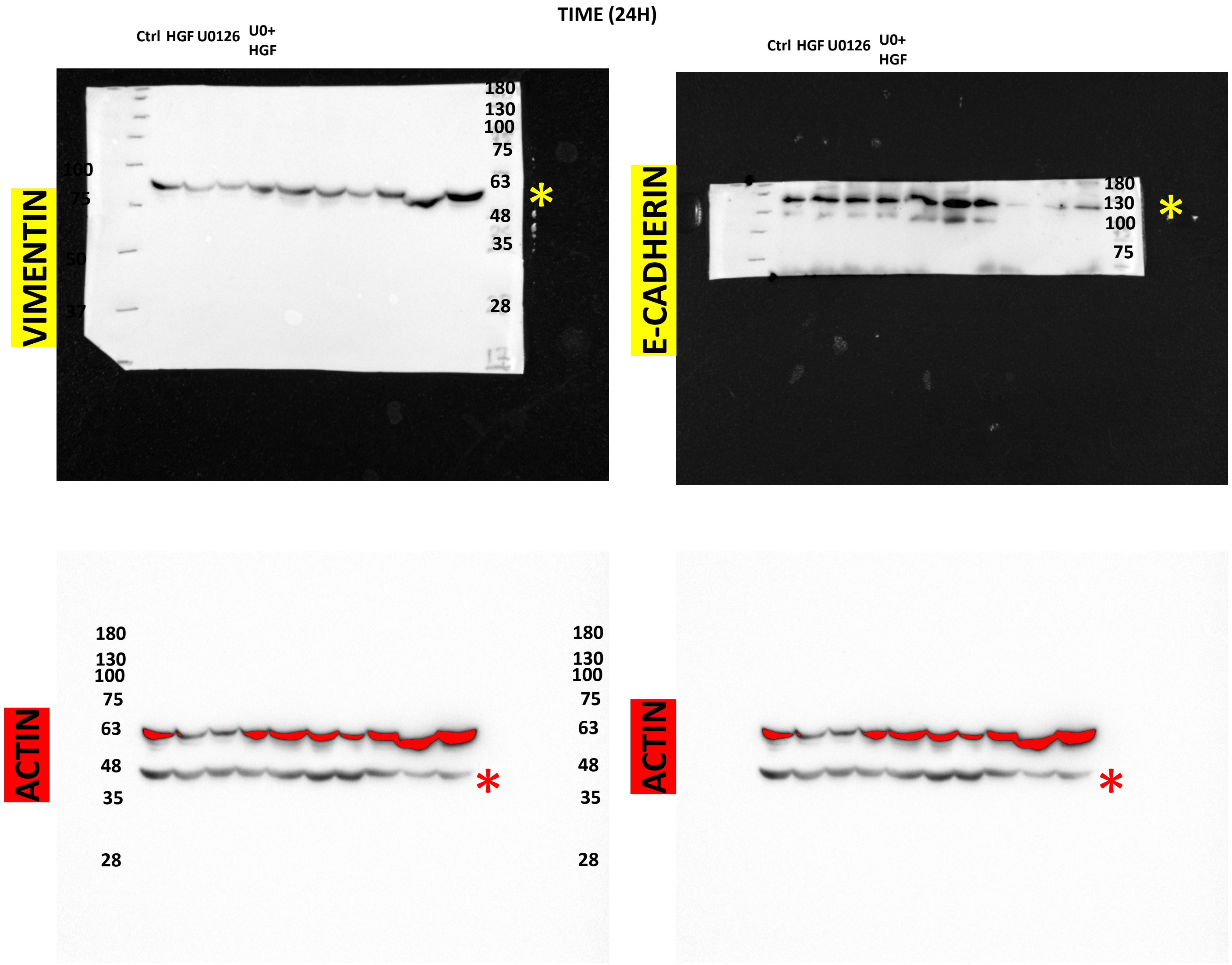

These blots are relative to results presented in FIGURE 8

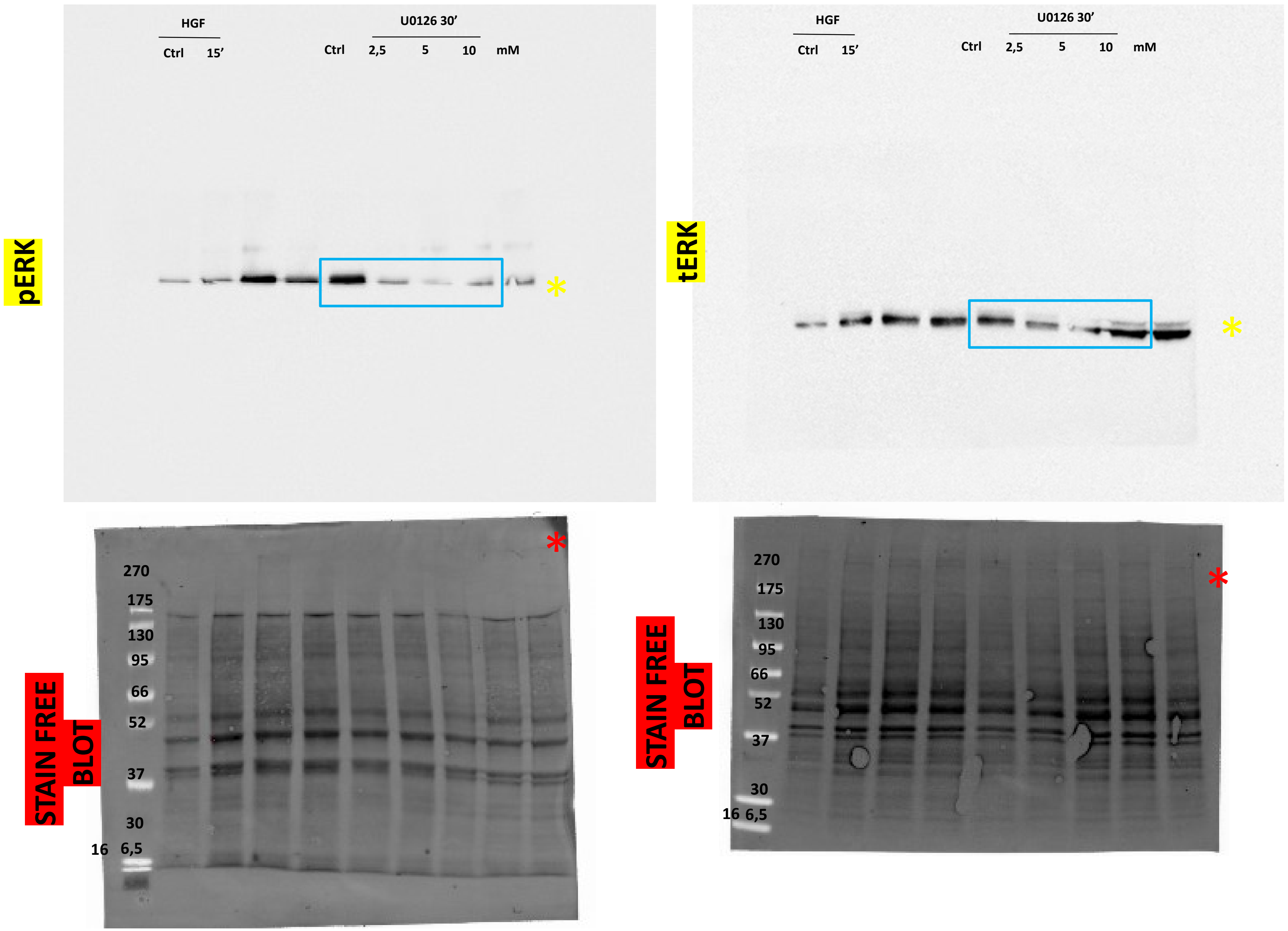

Blue boxes indicate WB lines selected for figures included in the paper.
Figure S1
